# Multi‐Enzyme Mimetic Molybdenum Nitride Nanozymes Reshape Subgingival Microenvironment for Synergistic Periodontitis Therapy via ROS Regulation and Microbiome Remodeling

**DOI:** 10.1002/advs.202517770

**Published:** 2026-04-09

**Authors:** Weiyu Zhang, Zhongwei Yang, Yunfan Zhang, Longwei Wang, Xiaoyu Zhang, Jiahui Mao, Yizhi Dai, Yifan Yuan, Mingge Wang, Xin Yang, Xin Yu, Jing Liu, Chunying Chen

**Affiliations:** ^1^ Department of Orthodontics Peking University School and Hospital of Stomatology & National Center for Stomatology & National Clinical Research Center for Oral Diseases & National Engineering Research Center of Oral Biomaterials and Digital Medical Devices& Beijing Key Laboratory of Digital Stomatology & NHC Key Laboratory of Digital Stomatology & NMPA Key Laboratory for Dental Materials Beijing P. R. China; ^2^ Department of Orthodontics Peking University School and Hospital of Stomatology Beijing P. R. China; ^3^ CAS Key Laboratory for Biomedical Effects of Nanomaterials and Nanosafety and CAS Center for Excellence in Nanoscience National Center for Nanoscience and Technology of China Beijing P. R. China; ^4^ Key Laboratory of Resource Biology and Biotechnology in Western China Ministry of Education College of Life Sciences Northwest University Xi'an P. R. China; ^5^ Department of Geriatric Dentistry Peking University School and Hospital of Stomatology Beijing P. R. China; ^6^ Institute for Advanced Interdisciplinary Research (iAIR) School of Chemistry and Chemical Engineering University of Jinan Jinan P. R. China

**Keywords:** Mo_5_N_6_ nanozymes, multi‐enzyme activity, oral microbiota, periodontitis, ROS regulation

## Abstract

Periodontitis, a chronic inflammatory disease initiated and sustained by plaque microorganisms and host immune response, remains an intractable oral disease and a leading cause of tooth loss worldwide. Traditional mechanical debridement and adjunctive antibiotic or antiseptic therapy often shows limited efficacy due to the complex anatomical structure, concerns regarding antimicrobial resistance, and poor penetration and retention within the subgingival infection niche. To overcome this limitation, we designed a Mo‐N coordinated nanozyme exhibiting synergistic mimetic activities of multiple enzymes, including peroxidase (POD)‐like, oxidase (OXD)‐like, and catalase (CAT)‐like activity. Benefiting from Mo‐N coordination and multi‐enzyme mimetic behavior, Mo_5_N_6_ nanozymes dynamically modulate local oxidative reactions within the gingival sulcus, thereby effectively damaging pathogenic bacteria while avoiding excessive oxidative stress. The nanozymes efficiently suppress anaerobic Gram‐negative periodontal pathogens sensitive to elevated reactive oxygen species (ROS), facilitating efficient attenuation of pathogenic stimuli. This strategy not only enhances the periodontal microenvironment but also facilitates the restoration of commensal microbiota and regeneration of periodontal tissues, highlighting the therapeutic potential of Mo_5_N_6_ nanozymes in periodontitis treatment.

## Graphical abstract

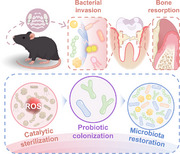

## 1 Introduction

Periodontitis is a prevalent chronic inflammatory disease of the tooth‐supporting tissues, characterized by periodontal pocket formation, progressive alveolar bone loss, and eventual tooth loss, impairing mastication and quality of life [1, 2, 3]. Beyond a localized oral condition, it is strongly linked to systemic disorders, including cardiometabolic, neurodegenerative, and autoimmune diseases [4, 5, 6]. The disease arises from a shift to dysbiotic oral microbiota, where keystone pathogens such as *Porphyromonas gingivalis* (Pg) disrupt host‐microbe homeostasis and immune regulation. This fosters a polymicrobial community that perpetuates inflammation, accelerates tissue destruction, and creates nutrient‐rich niches for opportunistic pathogens [7, 8, 9]. Mechanical debridement, including scaling and root planing, remains the clinical cornerstone for periodontitis treatment. However, due to the complex anatomy of periodontal pockets and the persistence of structured bacterial communities, adjunctive antimicrobial strategies are frequently employed to enhance bacterial clearance [10, 11]. Among these, hydrogen peroxide (H_2_O_2_) irrigation has been widely used in clinical practice owing to its broad‐spectrum antibacterial activity and accessibility. Nevertheless, the clinical efficacy of H_2_O_2_ is fundamentally constrained by its nonspecific diffusion, limited penetration into the subgingival infection niche, and rapid clearance by saliva. Moreover, clinical high‐dose of H_2_O_2_ (3%) increases the risk of mucosal damage and microbial imbalance, and, in rare cases, can cause adverse events such as gas embolism [12, 13]. These limitations highlight the urgent need for new adjunctive antimicrobial strategies with enhanced efficiency and safety that disrupt the persistent infectious microenvironment, preserve oral microbial homeostasis, and break the self‐sustaining cycle of dysbiosis and inflammation. In this context, generating elevated yet spatially confined and dynamically regulated reactive oxygen species (ROS) at the site of infection represents a promising strategy. Unlike bolus administration of concentrated H_2_O_2_, localized catalytic ROS generation enables antibacterial efficacy to be achieved at substantially lower overall oxidant exposure, thereby reducing collateral damage to surrounding tissues while preserving antimicrobial performance.

Nanozymes with intrinsic enzyme mimicking catalytic activity have emerged as compelling alternatives to natural enzymes owing to their high stability, tunable activity, and cost‐effective synthesis [14, 15, 16]. Since their first report in 2007, nanozymes have found widespread applications in biosensing, environmental remediation, and antimicrobial therapy, particularly in scenarios where conventional enzymes are hampered by poor stability, high production costs, and stringent operational requirements [17, 18, 19, 20, 21]. Notably, the pH‐dependent catalytic feature of nanozymes aligns well with the pathological characteristics of periodontitis marked by bacterial infection and a persistently dysregulated local inflammatory milieu. Accordingly, nanozyme‐based strategies have been increasingly explored for periodontitis management. Through peroxidase (POD)‐like, oxidase (OXD)‐like, catalase (CAT)‐like, or superoxide dismutase‐like activities, nanozymes can effectively decompose endogenous substrates such as H_2_O_2_ to eliminate pathogens [22, 23] and alleviate excessive oxidative stress in infection‐associated inflammation [24, 25]. Increasingly, multi‐enzyme nanozymes that integrate complementary catalytic functions have been proposed to balance effective microbial control with redox homeostasis [26, 27, 28, 29]. However, most reported nanozymes still suffer from poor adaptability to the complex inflammatory microenvironment and limited catalytic efficiency under physiological conditions, which hinder their long‐term therapeutic efficacy [30, 31]. These challenges have prompted increasing efforts to identify catalytic platforms that combine structural stability with flexible redox regulation.

Transition metal‐based nanozymes, including oxides, sulfides, and metal‐nitrogen coordination frameworks, have been widely explored for antibacterial and inflammation‐related therapies through catalytic regulation of redox reactions [32]. Among them, transition metal nitride (TMN) nanozymes, inspired by M‐N_x_ active centers in natural metalloenzymes, have attracted increasing attention owing to their favorable physicochemical properties and catalytic robustness. Incorporation of nitrogen into metal lattices modulates the electronic structure and enhances substrate adsorption and activation, thereby improving catalytic efficiency in redox reactions [33, 34]. Representative TMN systems such as TiN and WN have demonstrated enhanced POD‐like activity and H_2_O_2_ decomposition efficiency compared with their oxide or sulfide counterparts, highlighting the catalytic advantages of M‐N coordination [35, 36]. Compared with other TMN nanozymes, molybdenum‐based nitrides are particularly attractive for biomedical catalysis due to the flexible redox chemistry and favorable biocompatibility of molybdenum [37]. In contrast, commonly studied molybdenum sulfides such as MoS_2_ exhibit catalytic activity that strongly depends on defect density and external activation and are prone to surface oxidation or sulfur loss under physiological conditions, which compromises long‐term stability [38]. Although nitrogen‐doped MoS_2_ (N‐MoS_2_) partially improves catalytic activity through surface electronic modulation, its catalytic behavior remains largely confined to surface modification [39]. By comparison, fully nitrided Mo‐based materials form continuous Mo‐N coordination frameworks that dominate the crystal structure, enabling stabilization of multiple Mo oxidation states and reversible redox cycling during catalytic reactions. Such coordination‐driven architecture provide enhanced structural integrity, chemical stability, and catalytic continuity in reactive inflammatory environments. As a representative fully nitrided molybdenum nitride, Mo_5_N_6_ can therefore be regarded as an advanced Mo‐N‐based nanozyme platform with improved catalytic adaptability and therapeutic potential for periodontitis treatment [40, 41].

**SCHEME 1:**
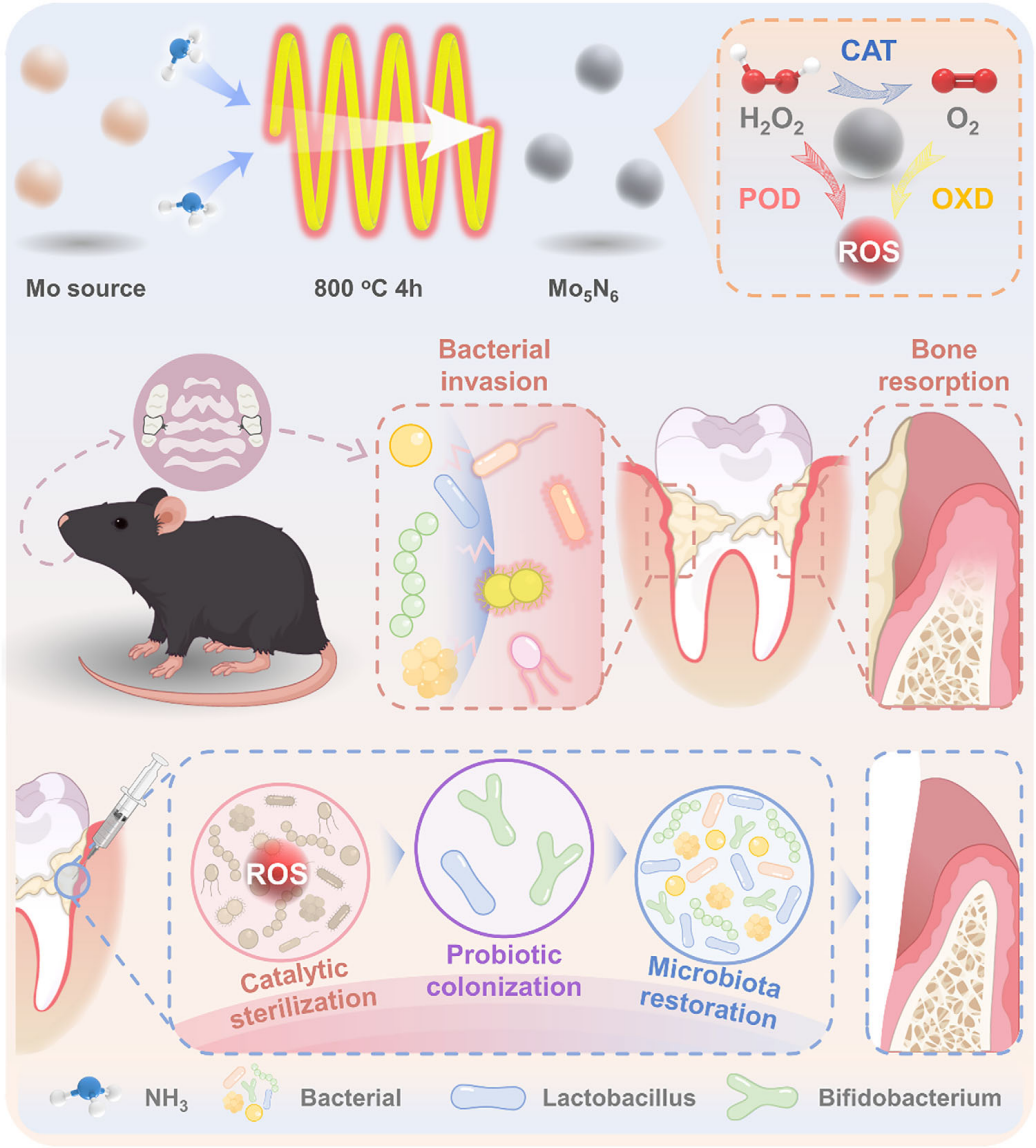
Schematic illustration of the synthesis of Mo_5_N_6_ nanozymes and its therapeutic mechanism against periodontitis.

In this study, we developed a molybdenum nitride (Mo_5_N_6_) nanozyme platform with multi‐enzyme catalytic activities, enabling coordinated regulation of infection, inflammation, and tissue repair in the periodontal microenvironment [42, 44]. Leveraging its Mo‐N active centers, the material catalyzes H_2_O_2_ and O_2_ within the gingival sulcus to generate ROS in a spatially confined manner. In addition, Brunauer‐Emmet‐Teller (BET) analysis revealed the high specific surface area and mesoporous structure of Mo_5_N_6_ nanoparticles, which increase active‐site accessibility and enhance substrate diffusion [45, 46]. The resulting ROS efficiently eradicates subgingival pathogens, thereby vacating ecological niches that favor early recolonization by commensal oral bacteria [47]. Concurrently, the OXD‐like activity enhances antibacterial efficacy by oxidizing bacterial substrates. Subsequently, the substrate‐concentration‐dependent attenuation of POD‐like activity, in concert with CAT‐like activity, efficiently decomposes excess H_2_O_2_ into O_2_ and H_2_O, thereby dynamically alleviating oxidative stress and minimizing collateral damage to healthy tissues [48, 49, 50]. Such multi‐catalytic synergism enables precise modulation of local ROS levels: on the one hand, suppressing the recolonization of Gram‐negative pathogens highly sensitive to low‐dose ROS, and on the other, creating a favorable ecological niche for commensal microbiota, thereby steering microbial succession toward a symbiotic state. The microbiota shift breaks the vicious cycle between the dysbiotic community and immune disorder [51]. Reduction of periodontal pathogenic factors and recovery of probiotics collectively downregulate proinflammatory cytokines, repolarize macrophages and stimulate anti‐inflammatory mediators, ultimately restoring periodontium structural and functional integrity [52, 53]. (Scheme 1). In a periodontitis model, Mo_5_N_6_ treatment markedly promoted soft tissue repair, suppressed alveolar bone resorption, and reduced inflammatory marker levels. Subgingival microbiome profiling revealed that both α‐ and β‐diversity indices approached those observed in healthy controls. Such microbiota reconfiguration is expected to lower the risk of disease recurrence by preventing the resurgence of pathogenic taxa. Collectively, we establish a synergistic “ROS‐mediated bactericidal and microbiota‐modulatory” strategy for periodontitis management.

## 2 Results and Discussion

### 2.1 Synthesis and Characterization of Mo_5_N_6_ Nanozymes

**FIGURE 1:**
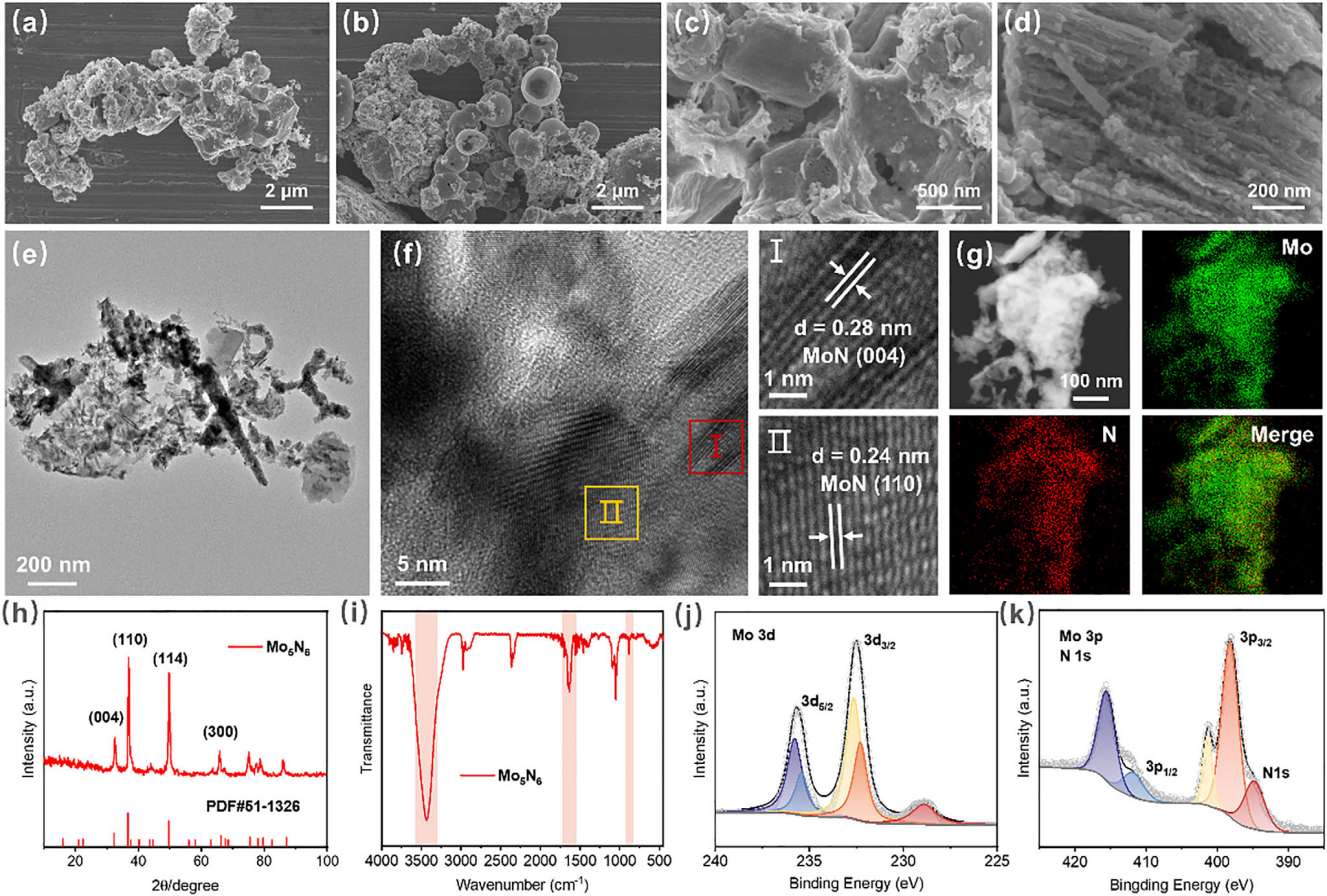
Synthesis and characterization of Mo_5_N_6_. (a‐d) SEM (e) TEM, (f) HRTEM and (g) EDS images of Mo_5_N_6_. (h) XRD spectra of Mo_5_N_6_ and the standard spectra of Mo_5_N_6_ (i) FTIR spectra of Mo_5_N_6_. (j) Mo 3d and (k) N1s XPS spectrum of Mo_5_N_6_.

To obtain multi‐enzyme mimetic nanomaterials, Mo_5_N_6_ nanozymes were synthesized through a one‐step annealing of a molybdenum precursor. Scanning electron microscopy (SEM) images show that the resulting particles are densely packed and amorphous, with irregular morphologies (Figure 1a–d). Transmission electron microscopy (TEM) displays lattice fringes with spacings of 0.28 and 0.24 nm, corresponding to the (004) and (110) planes of Mo_5_N_6_ (Figure 1e,f). Energy‐dispersive X‐ray spectroscopy (EDS) mapping demonstrates a uniform distribution of molybdenum and nitrogen across the sample, along with successful nitrogen incorporation (Figure 1g). X‐ray diffraction (XRD) confirms the phase identity, with distinct reflections at 32.32°, 36.71°, 49.71°, and 66.11° indexed to the (004), (110), (114), and (300) planes of hexagonal Mo_5_N_6_, consistent with XRD standard card (Figure 1h). Fourier‐transform infrared (FTIR) spectra exhibit characteristic absorption bands at 3427, 1631, 1047, and 590 cm^−1^, corresponding to N‐H stretching, bending, in‐plane rocking, and Mo‐N stretching vibrations, respectively (Figure 1i), supporting the presence of Mo‐N bonding. The presence of Mo and N can be observed in the X‐ray photoelectron spectroscopy (XPS) full spectrum (Figure S1). As shown in the high‐resolution Mo 3d XPS spectrum (Figure 1j), the Mo species mainly exist in mixed oxidation states of Mo^4+^ and Mo^6+^. Among them, Mo^6+^ is the dominant component, with a relative atomic fraction of 64.4% (Figure S2). The high proportion of Mo^6+^ suggests a surface highly oxidized electronic structure, which favors H_2_O_2_ activation and thus enhances the POD‐like catalytic performance. In the N 1s and Mo 3p regions, peaks at 398.25 and 394.8 eV confirm the formation of Mo‐N coordination bonds (Figure 1k). Nitrogen adsorption/desorption isotherms show a steep rise in uptake at high relative pressures (P/P_0_), suggesting the presence of interparticle porosity or mesoporous features. The BET surface area is measured to be 210.3 ± 3.6 m^2^/g, offering ample accessible surface for catalytic activity (Figure S3). Barrett‐Joyner‐Halenda (BJH) analysis further reflects the characteristics of interparticle gaps formed by nanoparticle aggregation (Figure S4).

### 2.2 Characterization of the Multi‐Enzyme‐Mimicking Catalytic Activity of Mo_5_N_6_

**FIGURE 2:**
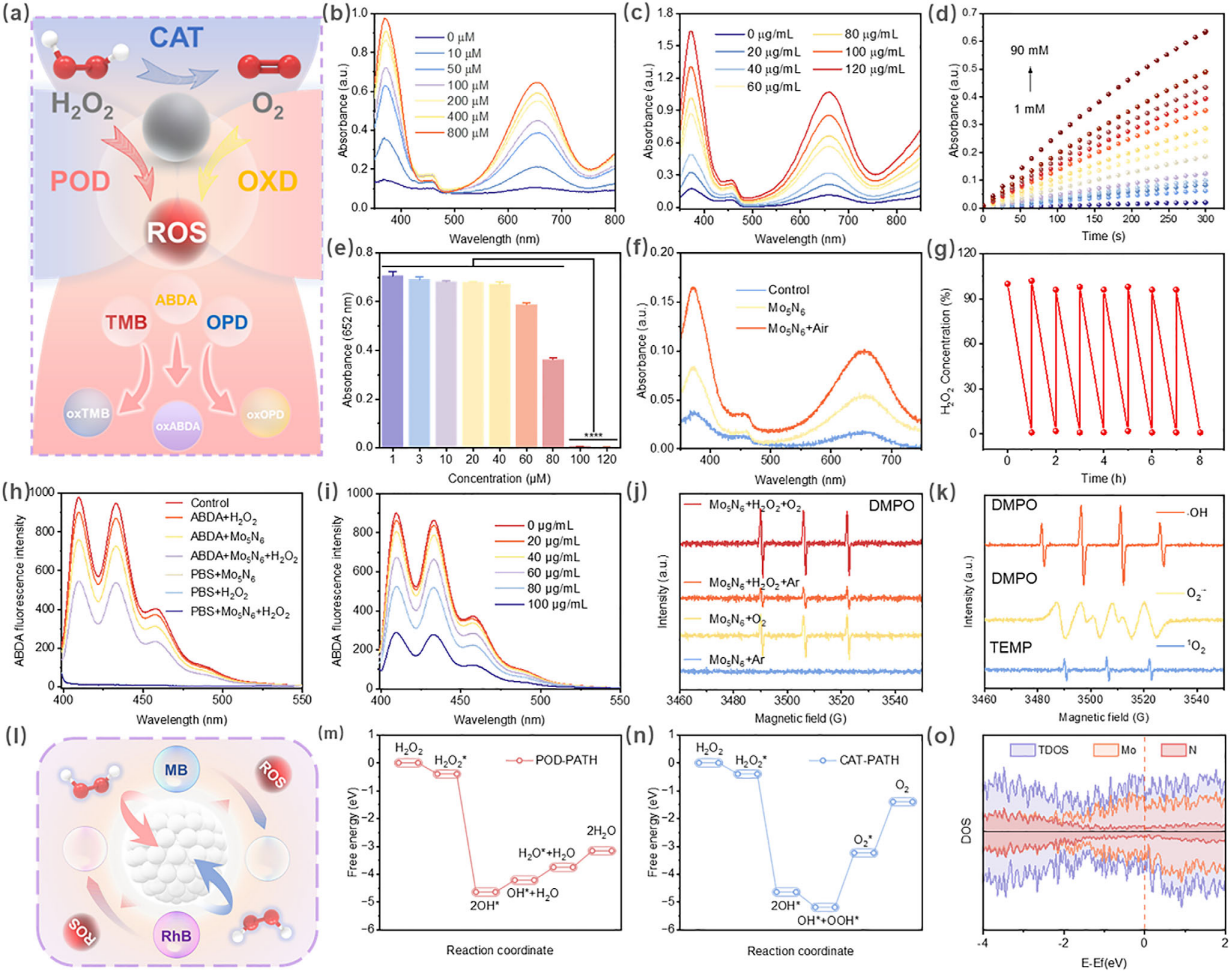
ROS‐production performances of Mo_5_N_6_ nanozymes. (a) Reaction scheme for H_2_O_2_ decomposition and reaction scheme for TMB, ABDA, and OPD oxidation. Absorbance of oxTMB with different (b) H_2_O_2_ and (c) Mo_5_N_6_ concentrations. (d) The time‐dependent absorbance changes at 652 nm for the POD‐like activity of Mo_5_N_6_ under varying TMB concentrations. (e) Endpoint absorbance at 652 nm upon addition of varying concentrations of AA. (f) UV–Vis spectra of oxTMB generated via POD‐like activity under varying reaction conditions. (g) Quantified H_2_O_2_ consumption during cycle stability testing (a catalytic cycle was defined as one complete oxidation‐reduction reaction H_2_O_2_ under constant catalyst loading with fresh H_2_O_2_ added after each run). Mo_5_N_6_ retained 96.1% activity after nine consecutive cycles. UV‐vis spectra of ABDA under (h) varying reaction conditions and (i) graded concentrations. EPR spectra of (j) ^1^O_2_ generated under controlled catalytic conditions and (k) ·OH, O_2_^·−^ and ^1^O_2_ using different spin‐trapping agents: DMPO was used for •OH and O_2_^·−^ detection, while 2,2,6,6‐Tetramethylpiperidine (TEMP) was used for ^1^O_2_ detection. (l) Schematic illustration of MB and RhB degradation. (m) Free energy profile of the POD‐like reaction pathway, illustrating H_2_O_2_ adsorption, O‐O bond cleavage, and *OH‐involved intermediates. (n) Free energy profile of the CAT‐like reaction pathway, showing the stepwise disproportionation of H_2_O_2_ into O_2_ and H_2_O via *OOH/*O_2_ intermediates. (o) Total density of states (TDOS) and projected density of states (PDOS) of Mo d and N p orbitals, with the Fermi level set to 0 eV (dashed line). The significance of the data was calculated by the one‐way ANOVA. ns: no significance, ^*^ *p* < 0.05, ^**^ *p* < 0.01, ^***^ *p* < 0.001, ^****^ *p* < 0.0001.

Subsequently, the enzyme‐like catalytic activity of Mo_5_N_6_ was investigated (Figure 2a). We first examined the POD‐like activity of Mo_5_N_6_ in catalyzing H_2_O_2_ to generate ·OH. 3,3′,5,5′‐Tetramethylbenzidine (TMB) and o‐phenylenediamine (OPD) were employed as chromogenic substrates to monitor the catalytic reaction. The ·OH radicals generated by Mo_5_N_6_ catalysis converted TMB into oxTMB, which exhibits a characteristic absorption peak at 652 nm. As the concentrations of TMB, Mo_5_N_6_, or H_2_O_2_ increased, a significant increase in the absorption peak at 652 nm was observed (Figure 2b–d). Similar results were obtained when OPD was used as the substrate (Figure S5). Moreover, the POD‐like activity of Mo_5_N_6_ was significantly superior to that of MoO_3_, MoS_2_ (Figure S6), TiN and WN (Figure S7). Subsequently, ascorbic acid (AA) was introduced into the system to suppress the Mo_5_N_6_‐catalyzed oxidation of TMB, and it was found that only at high AA concentrations could the catalytic activity of Mo_5_N_6_ be inhibited, indicating its excellent catalytic performance (Figure 2e). Meanwhile, when air was introduced into the system without H_2_O_2_, the catalytic activity of Mo_5_N_6_ was further enhanced, suggesting that, in addition to POD‐like activity, Mo_5_N_6_ also exhibits OXD‐like activity (Figure 2f). Furthermore, we performed cyclic stability tests and catalytic condition screening for Mo_5_N_6_ and found that Mo_5_N_6_ maintained excellent catalytic activity over a broad range of pH and temperatures (Figure S8). Moreover, no significant decline in performance was observed after multiple catalytic cycles, which is favorable for sustaining Mo_5_N_6_’s catalytic stability in complex microenvironments (Figure 2g). In addition, when 9,10‐anthracenediyl‐bis(methylene)dimalonic acid (ABDA) was used as the substrate, we evaluated the singlet oxygen (^1^O_2_) generation capability of Mo_5_N_6_ under various catalytic conditions. It was observed that even in the absence of H_2_O_2_, Mo_5_N_6_ could induce a decrease in ABDA fluorescence intensity, and the fluorescence intensity decreased further upon the addition of H_2_O_2_, indicating that Mo_5_N_6_ possesses OXD‐like activity capable of converting O_2_ into ^1^O_2_, and that its CAT‐like activity in the presence of H_2_O_2_ could further enhance this OXD‐like functionality (Figure 2h,i; Figure S9). CAT‐like activity is markedly suppressed under acidic conditions though (Figure S10). The electron paramagnetic resonance (EPR) spectroscopy results further confirmed the above findings (Figure 2j). Additionally, 5,5‐dimethyl‐1‐pyrroline‐N‐oxide (DMPO) was used as a spin‐trapping agent, and characteristic ·OH signals were detected under the presence of H_2_O_2_, with a quartet signal intensity ratio of 1:2:2:1, and the formation of superoxide radicals (O_2_^·−^) was also observed in the EPR spectra. Furthermore, steady‐state kinetic analysis of Mo_5_N_6_ was conducted using the Lineweaver‐Burk double‐reciprocal plot method, yielding a *K*_m_ value of 0.075 mM and a *V*_max_ of 12.64 × 10^−6^ M·s^−1^, indicating that Mo_5_N_6_ exhibits excellent H_2_O_2_ affinity and superior catalytic activity compared not only to natural horseradish peroxidase (HRP) but also to many other reported studies (Figure S11, Tables S1 and S2) [54]. The degradation of methylene blue (MB) and rhodamine B (RhB) by Mo_5_N_6_ in the presence of H_2_O_2_, further validated its outstanding enzyme‐like catalytic activity (Figure 2l; Figure S12).

Density functional theory (DFT) calculations were conducted to compare the free energy evolution of H_2_O_2_ decomposition on the Mo_5_N_6_ surface along the POD‐like and CAT‐like pathways. Along the POD‐like pathway, adsorbed H_2_O_2_ undergoes facile O‐O bond cleavage to generate *OH intermediates with a pronounced exothermic energy decrease, indicating a strong thermodynamic preference for ·OH formation and supporting POD‐like activity under acidic conditions relevant to periodontal infection sites. In contrast, along the CAT‐like pathway, H_2_O_2_ preferentially proceeds through *OOH/*O_2_ intermediates and is gradually disproportionated into O_2_ and H_2_O with relatively low energy barriers and a smoother free energy landscape, suggesting efficient radical‐free H_2_O_2_ decomposition under neutral to mildly alkaline conditions (Figure 2m, n and S13). Density of states (DOS) analysis reveals pronounced hybridization between Mo d and N p orbitals near the Fermi level, which increases surface electronic density, facilitates multielectron transfer, and lowers the energetic barriers for H_2_O_2_ adsorption and activation. Collectively, these results demonstrate that Mo_5_N_6_ enables environment‐dependent switching between POD‐ and CAT‐like reaction pathways through modulation of reaction intermediate stability and electronic structure, providing a theoretical basis for its effective ROS regulation in periodontitis therapy (Figure 2o).

Finally, minimal changes in morphology, size and surface potential were shown after immersion in ultrapure water and PBS (pH 7.4) for 5 h measured by TEM images, dynamic light scattering (DLS), and Zeta potential, indicating good colloidal stability of Mo_5_N_6_ (Figure S14a–d). Furthermore, we conducted TEM characterization of the material before and after the POD‐like catalytic reaction, and the results show that the lattice structure of Mo_5_N_6_ nanozymes remains intact during H_2_O_2_ decomposition, with no obvious morphological changes observed, demonstrating excellent chemical and structural stability (Figure S14d,e).

### 2.3 In Vitro Antibacterial Performance of Mo_5_N_6_

Pg and *Fusobacterium nucleatum* (Fn), recognized as key pathogens in periodontitis progression, were selected as representative bacterial models to assess the in vitro antibacterial performance of our nanozymes [55, 56]. Capitalizing on the remarkable multi‐enzyme‐mimicking properties and high H_2_O_2_ affinity of Mo_5_N_6_, its bactericidal efficacy was systematically examined under controlled experimental conditions. We selected 3% hydrogen peroxide as the positive control in our experiments named active treatment (AT) group. This concentration is commonly used in clinical periodontitis management as an adjunctive therapy following mechanical debridement but also suffers from concerns about the potent tissue damage and inflammation at relevantly high concentrations. In this study, it served as a reference to evaluate the antimicrobial and tissue‐protective effects of Mo_5_N_6_ combined with low‐dose H_2_O_2_, highlighting the possible detoxifying and synergistic enhancement properties of our combined therapy. 200 µM H_2_O_2_ and 100 µg mL^−1^ Mo_5_N_6_ were used separately or simultaneously in other experimental groups, based on OD_600_ measurement across Pg cultures treated with varying concentrations of Mo_5_N_6_ and different incubation periods (Figure S15).

**FIGURE 3:**
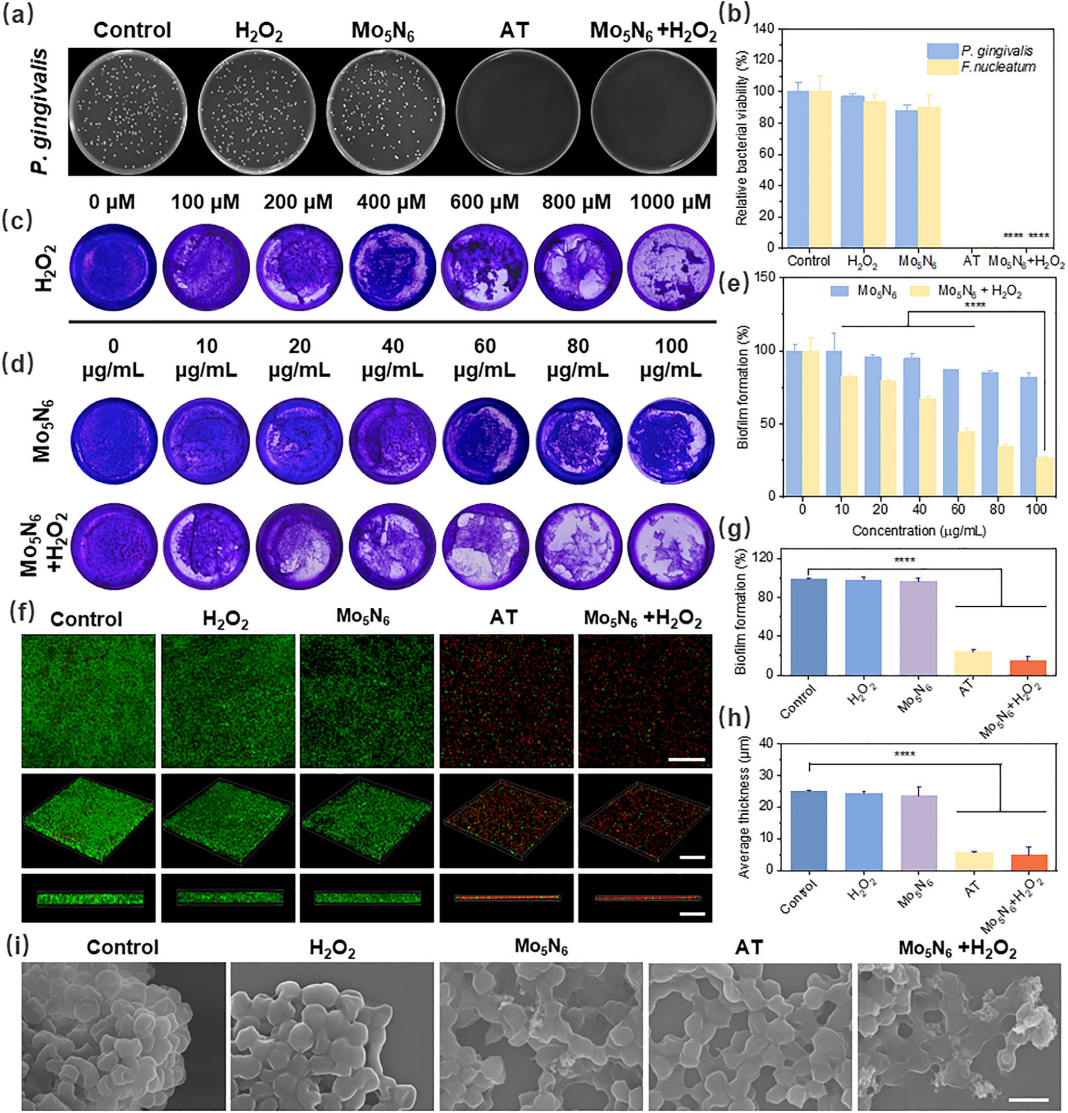
In vitro antibacterial performance evaluation of Mo_5_N_6_ on Pg. Comparison of Pg across five groups was shown: the untreated control group (Control), the group exposed to 200 µM H_2_O_2_ (H_2_O_2_) or 100 µg mL^−1^ Mo_5_N_6_ alone (Mo_5_N_6_), the group exposed to 3% H_2_O_2_ (active treatment, AT) and the group receiving a combined treatment of 100 µg mL^−1^ Mo_5_N_6_ and 200 µM H_2_O_2_ (Mo_5_N_6_ + H_2_O_2_). (a) Photographs of bacterial colonies formed by Pg. (b) Relative bacteria viabilities of Pg determined by plate count method. (c) Crystal violet staining images of bacterial biofilms after treatment with different H_2_O_2_ concentrations. (d) Crystal violet staining images of Pg biofilms under different material concentrations with/without H_2_O_2_ treatment. (e) The biomass of Pg biofilms was quantified by crystal violet staining at OD_595_. (f) 3D CLSM Live/Dead staining images of Pg biofilms after different treatments (scale bar: 50 µm). Statistical analysis of (g) biofilm biomass and (h) average thickness in Panel (f). (i) SEM images of Pg (scale bar: 1 µm). The data are presented as the mean ± SD of *n* = 3. The significance of the data was calculated by the one‐way ANOVA. ns: no significance, ^*^ *p* < 0.05, ^**^ *p* < 0.01, ^***^ *p* < 0.001, ^****^ *p* < 0.0001.

The survival rate of planktonic bacteria under different treatments was then assessed using classical plate colony counting assays. As shown in Figure 3a and Figure S16a, the Mo_5_N_6_ + H_2_O_2_ treatment achieved pronounced antibacterial effects against Pg and Fn, showing no statistically significant difference from the positive control group. Whereas treatment with 200 µM H_2_O_2_ or Mo_5_N_6_ alone resulted in only limited bacteriostatic effects (Figure 3b; Figure S16c). Disruption of the bacterial membrane leads to the release of intracellular contents into the extracellular environment. To further evaluate the extent of bacterial membrane damage, nucleic acid and protein leakage assays were conducted. As shown in Figure S17a, the Mo_5_N_6_ + 200 µM H_2_O_2_ and the 3% H_2_O_2_ group displayed a significantly higher OD_260_ value compared to other groups, indicating more extensive nucleic acid leakage and thus stronger antibacterial activity. Similarly, results from the bicinchoninic acid (BCA) protein assay revealed analogous trends in protein release (Figure S17b). Moreover, the interaction between Mo_5_N_6_ and the bacterial membrane was corroborated by assessing membrane permeability using o‐nitrophenyl‐β‐D‐galactopyranoside (ONPG). The findings demonstrated a marked increase in membrane permeability of Pg in the Mo_5_N_6_ + 200 µM H_2_O_2_ and the 3% H_2_O_2_ group (Figure S17c). Hence, Mo_5_N_6_ in combination with low‐dose H_2_O_2_ demonstrated remarkable antibacterial activity, effectively disrupting bacterial structures, which is critical for efficient bacterial killing and the elimination of resilient periodontal pathogens.

Single‐strain mature biofilms of Pg and Fn were established to evaluate the bactericidal and biofilm‐penetration capabilities of the nanozymes, as well as the resulting changes in biofilm architecture after treatment. Crystal violet staining was used to quantitatively assess the structural integrity of Pg/Fn biofilms under varying Mo_5_N_6_ concentrations, with or without the presence of H_2_O_2_. In the presence of H_2_O_2_ alone, disruption of biofilm integrity required relatively high concentrations (Figure 3c; Figure S18). However, in the presence of Mo_5_N_6_, a significant Mo_5_N_6_ dose‐dependent biofilm clearance effect against Pg and Fn was observed (Figure 3d; Figure S16b). At a Mo_5_N_6_ concentration of 100 µg mL^−1^, biofilm clearance rates of 73.00% for Pg and 77.94% for Fn were achieved (Figure 3e; Figure S16c). Live/Dead fluorescence staining of bacterial viability was further employed to evaluate the disruptive effect of Mo_5_N_6_ on mature Pg and Fn biofilms. The blank control group displayed dense, intact biofilms composed predominantly of live bacteria, whereas the AT group showed abundant dead bacteria. Similar to the blank group, the low H_2_O_2_‐ and Mo_5_N_6_‐alone treatments exhibited high bacterial viability and intact structure. In contrast, the Mo_5_N_6_ + H_2_O_2_ treatment achieved the highest biofilm eradication rates, 85.71% for Pg and 90.38% for Fn, and reduced biofilm thickness from over 20 µm in controls to below 8 µm (Figure 3f–h; Figure S16d–f). These findings demonstrate that Mo_5_N_6_ possesses potent biofilm penetration and disruption capabilities under low H_2_O_2_ concentrations, making it promising for eliminating pathogens in deep periodontal pockets with persistent infections. Finally, SEM revealed that following Mo_5_N_6_ + H_2_O_2_ treatment, the nanozyme adhered to bacterial surfaces, inducing pronounced wrinkling and collapse (Figure 3i). Consistently, Mo_5_N_6_ exhibited potent in vitro activity against both Gram‐positive methicillin‐resistant *Staphylococcus aureus* (*MRSA*) and Gram‐negative chloramphenicol‐resistant *E. coli* (Chl^r^ *E. coli*), confirming that Mo_5_N_6_‐catalyzed ROS generation from H_2_O_2_ inflicted irreversible oxidative damage on bacteria (Figure S19).

### 2.4 In Vivo Antibacterial Performance and Therapeutic Efficacy of Mo_5_N_6_ in Periodontitis

**FIGURE 4:**
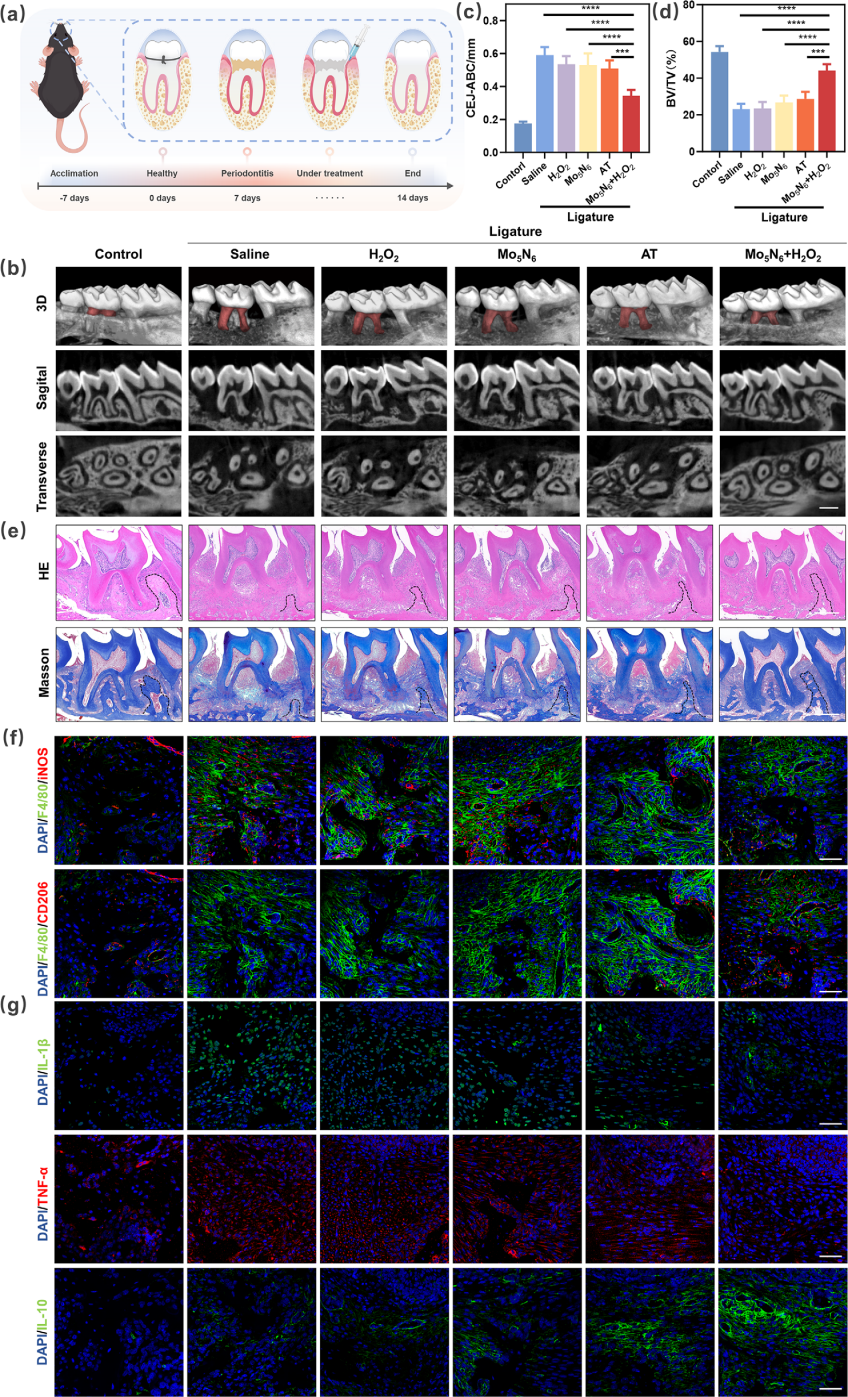
In vivo inhibition of periodontitis‐induced alveolar bone resorption by Mo_5_N_6_ intervention. (a) Schematic diagram of the ligature‐induced periodontitis model and the treatment process. (b) Representative 3D Reconstruction and 2D Micro‐CT visualization of the unilateral maxilla surrounding the second molar after treatment in mice (scale bar: 500 µm) (*n* = 8). (c) Distance from the CEJ to the ABC, indicating the extent of alveolar bone loss (*n* = 8). (d) Quantitative analysis of the bone volume fraction (BV/TV), reflecting the proportion of mineralized bone within the total tissue volume (*n* = 8). (e) H&E and Masson staining images of the periodontal tissue (scale bar: 400 µm). (f) Representative immunofluorescent staining of M1 and M2 macrophage markers in mouse periodontal tissues (scale bar: 50 µm) (*n* = 3). (g) Representative immunofluorescent staining of inflammatory factors in mouse periodontal tissues (scale bar: 50 µm) (*n* = 3). The data are presented as the mean ± SD. The significance of the data was calculated by the one‐way ANOVA. ns: no significance, ^*^ *p* < 0.05, ^**^ *p* < 0.01, ^***^ *p* < 0.001, ^****^ *p* < 0.0001.

To assess the in vivo antimicrobial performance of Mo_5_N_6_ nanozymes, a ligature‐induced murine periodontitis model was established. After 1 week of acclimatization, 5‐0 silk sutures were placed around the cervical region of the bilateral maxillary second molars for 7 days to induce localized periodontal inflammation. Subsequently, topical treatment was administered into the buccal and lingual gingival sulcus twice a day for 7 days, with animals randomly assigned to six groups: Control group (without ligature), Saline (ligature + saline), H_2_O_2_ (ligature + 200 µM H_2_O_2_), Mo_5_N_6_ (ligature + 100 µg mL^−1^ Mo_5_N_6_), AT (ligature + 3% H_2_O_2_) and Mo_5_N_6_ + H_2_O_2_ (ligature + 100 µg mL^−1^ Mo_5_N_6_ + 200 µM H_2_O_2_) (Figure 4a). Following treatment, gingival crevicular sites were sampled using sterile swabs for bacterial culture. Colony‐forming unit (CFU) quantification revealed a marked reduction in bacterial load in the Mo_5_N_6_ + H_2_O_2_ group, indicating effective suppression of pathogenic bacteria within the subgingival environment (Figure S20). After 7 days, the unilateral maxilla specimens were analyzed by micro‐computed tomography (micro‐CT) analysis. Reconstructed three‐dimensional coronal images confirmed the successful induction of bone loss in the periodontitis model group. Treatment group showed therapeutic effects at different levels. Quantitative measurement of the vertical distance from the cementoenamel junction (CEJ) to the alveolar bone crest (ABC) at six predefined sites (mesial, central, distal on both buccal and lingual sides) revealed substantial bone resorption in the Saline group, with CEJ‐ABC distances increasing by ∼400 µm relative to the Control group. Compared with the Saline group, neither the H_2_O_2_ group nor the Mo_5_N_6_ group showed significant differences in alveolar bone height loss. The positive control group exhibited only limited improvement, which was markedly inferior to that observed in the Mo_5_N_6_ + H_2_O_2_ group. Notably, Mo_5_N_6_ + H_2_O_2_ treatment substantially attenuated alveolar bone resorption, reducing the CEJ‐ABC distance by approximately 250 µm and thereby partially preserving bone height. Coronal sections further revealed noticeable bone regeneration surrounding the root surfaces in this group (Figure 4b,c). To evaluate bone microarchitecture, key parameters including bone volume/total volume (BV/TV) and trabecular thickness (Tb.Th) were analyzed. The Saline group exhibited a ∼60% decrease in BV/TV relative to the Control group, whereas the Mo_5_N_6_ + H_2_O_2_ group retained approximately 70% of bone volume, the highest among all treatment groups. Tb.Th values were also significantly preserved (*p* < 0.0001), further supporting the efficacy of this strategy in maintaining alveolar bone integrity (Figure 4d; Figure S21). While AT group, a concentration commonly employed in clinical irrigation, exerted moderate antibacterial effects, it failed to achieve comparable therapeutic outcomes. This limited efficacy may be attributed to the inadequate local availability of H_2_O_2_ within the subgingival niche and its transient retention caused by rapid salivary clearance, resulting in incomplete elimination of pathogenic bacteria and sustained inflammation. Moreover, the nonspecific diffusion of H_2_O_2_ throughout the gingival sulcus and oral cavity imposes excessive and spatially uncontrolled oxidative stress on periodontal tissues, which further compromises therapeutic outcomes by aggravating inflammation, inhibiting cell proliferation, and disrupting local redox homeostasis. In contrast, Mo_5_N_6_ nanozymes enable controlled ROS generation under physiologically relevant H_2_O_2_ concentrations through catalytic regulation within the subgingival microenvironment, achieving efficient antibacterial effects while minimizing collateral oxidative damage. This catalytically adaptive behavior, together with favorable biocompatibility, contributes to the superior therapeutic performance of Mo_5_N_6_ nanozymes.

Histometric analysis of the maxillae was conducted in the mesial, distal, and furcation regions of the second maxillary molars to assess pathological changes and inflammatory status in the gingival tissue. Hematoxylin and eosin (H&E) staining revealed normal alveolar bone architecture and intact marginal epithelium in the Control group. In the Saline group, marked destruction of the gingival epithelium and lamina propria along with extensive inflammatory cell infiltration was observed, accompanied by interproximal alveolar crest disruption and bone resorption indicated by the increased CEJ‐ABC distance. These collectively reflected a pronounced inflammatory state of the periodontium. The other three groups, excluding the Mo_5_N_6_ + H_2_O_2_ group, exhibited varying degrees of improvement, although these changes were relatively modest. In contrast, the Mo_5_N_6_ + H_2_O_2_ group exhibited a marked reduction in the CEJ‐ABC distance and attenuated inflammatory infiltration, consistent with the micro‐CT findings (Figure 4e; Figure S22a). Masson staining demonstrated that the Saline group displayed disorganized periodontal ligament fibers and substantial loss of collagen fibers around the alveolar bone, indicating structural damage. Only the Mo_5_N_6_ + H_2_O_2_ group manifested markedly restored fiber density with organized alignment resembling normal tissue (Figure 4e; Figure S22a). These findings suggest that the material may promote periodontal soft tissue repair and regeneration by modulating local inflammation. Given that periodontitis disrupts the balance between osteoblasts and osteoclasts, tartrate‐resistant acid phosphatase (TRAP) staining was further performed on periodontal tissues. As a marker of osteoclast activity, TRAP staining allows for direct visualization of osteoclast distribution and quantity along bone margins. Compared with the Saline group, the Mo_5_N_6_ + H_2_O_2_ group exhibited a significant reduction in dark red multinucleated osteoclasts, indicating that the combined treatment effectively inhibited osteoclast activation and aggregation, thereby slowing alveolar bone resorption and promoting bone stability and regeneration (Figures S22b and S23).

The interplay between host and microbes plays a central role in shaping local inflammatory responses and immune status. Uncontrolled infections generate harmful byproducts, upregulate pro‐inflammatory factors, and trigger excessive immune activation, thereby exacerbating tissue damage. To validate the potential of Mo_5_N_6_ in mitigating pathogen‐triggered inflammation, we first performed an in vitro macrophage stimulation assay. A 2‐hour pulse treatment was first applied to Pg, followed by a complete medium exchange to simulate the bacterial eradication and subsequent dynamic gingival crevicular fluid flushing process. After an additional 24 h of culture, the resulting supernatant was collected to stimulate the RAW264.7 murine macrophage cell line. Real‐time PCR (RT‐qPCR) analysis revealed that the expression levels of pro‐inflammatory cytokines (TNF‐α and CD86) were significantly downregulated in the Mo_5_N_6_ + H_2_O_2_ group compared to the group challenged with untreated Pg (Figure S24). The result indicated that the nanozyme could effectively suppress host inflammatory signaling by eradicating the source of microbial provocation, thereby fundamentally removing the persistent stimuli that drives the pro‐inflammatory phenotype. Encouraged by this finding, we further investigated whether such microbial modulation could drive local periodontal immunity remodeling in our periodontitis model. Therefore, F4/80 was used as a pan‐macrophage marker, while iNOS and CD206 served as specific biomarkers for classically activated (M1) and alternatively activated (M2) macrophages, respectively. In the Control group, both iNOS and CD206 expression levels remained low. However, following ligature treatment, iNOS expression was significantly upregulated, indicating the presence of local inflammation. After Mo_5_N_6_ + H_2_O_2_ treatment, iNOS expression was markedly downregulated, accompanied by an increase in CD206 expression. Semi‐quantitative analysis further supported these trends (Figure 4f; Figures S25 and S26a). Immunofluorescence staining for inflammatory cytokines provided deeper insight into the inflammatory status of the periodontal tissues. Following ligature induction, periodontitis was characterized by a significant upregulation of pro‐inflammatory cytokines IL‐1β and TNF‐α. While treatment with low‐concentration H_2_O_2_ or Mo_5_N_6_ alone showed no marked alleviation of this pro‐inflammatory state, the group receiving the combined therapy demonstrated a significant reduction in both IL‐1β and TNF‐α levels. Concurrently, a significant elevation in the anti‐inflammatory and pro‐repair cytokine IL‐10 was observed (Figure 4g; Figure S26b–d). Although the 3% H_2_O_2_ treatment demonstrated potent bactericidal effects, comprehensive analysis of macrophage polarization ratios and inflammatory cytokine levels revealed that the mitigation of periodontal inflammation in this group was substantially inferior to that achieved by the combination treatment group (Figure S26b–d). This finding further supports the premise that the tissue damage and pro‐inflammatory effects induced by excessive H_2_O_2_ likely partially offset the benefits derived from its antibacterial activity, a conclusion consistent with the trends observed in previous murine bone tissue parameters.

Altogether, these results indicate that Mo_5_N_6_ + H_2_O_2_ treatment could rectify microbial dysbiosis and subsequently reverse the inflammatory microenvironment and promote macrophage polarization toward the M2 phenotype, thereby contributing to the resolution of chronic periodontal inflammation and building foundations for tissue repair.

**FIGURE 5:**
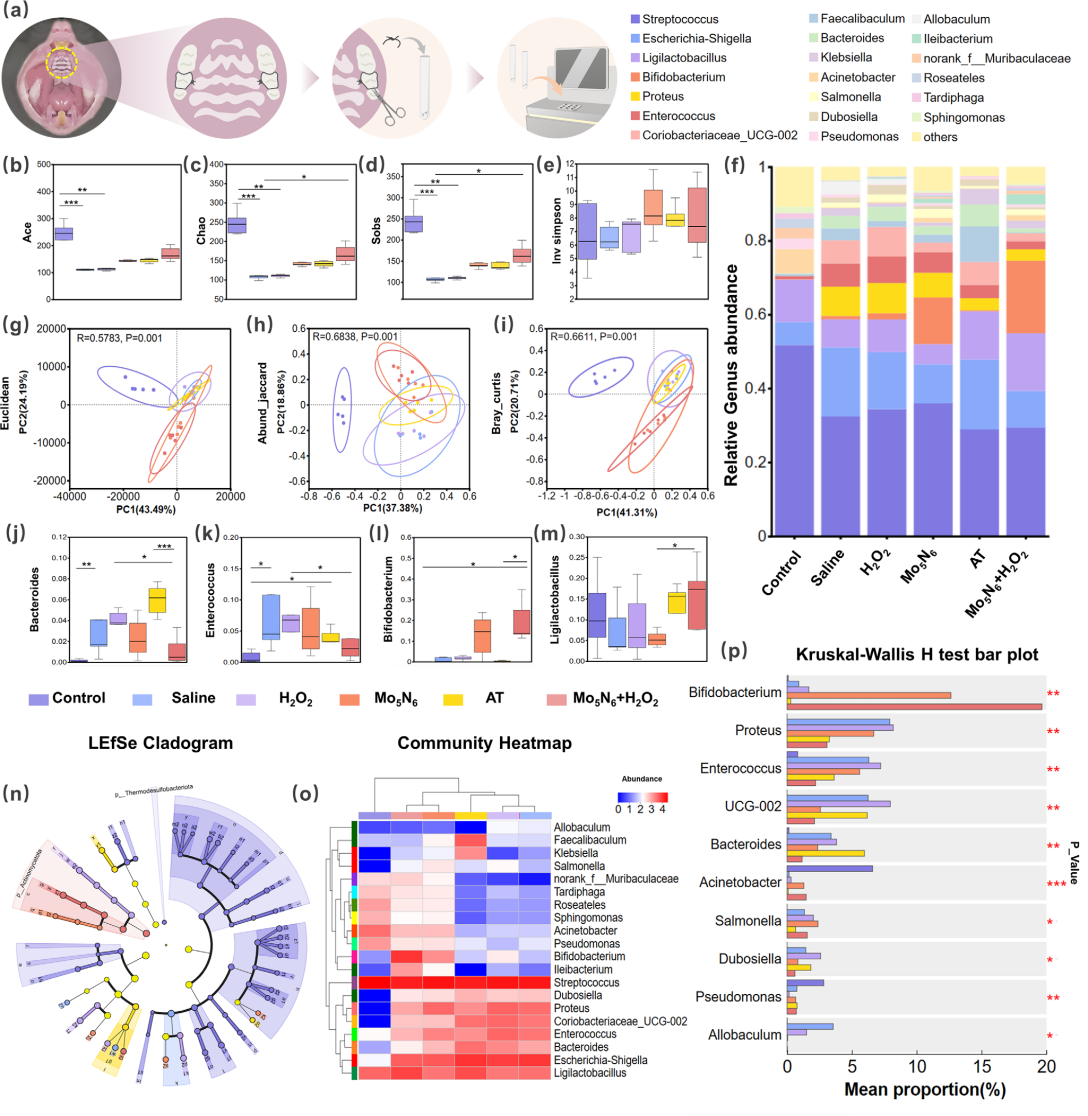
Regulatory effect of Mo_5_N_6_ treatment on the subgingival microbial community structure. (a) Schematic of sampling workflow for 16S rDNA analysis of the periodontal microbiome. (b–e) Analysis of α‐diversity of subgingival microbiome by Ace, Chao, Sobs and Inv simpson analyses (*n* = 5 for each group, Wilcoxon rank‐sum test). (f) Stacked boxplots of the relative abundance of subgingival microbial communities of each group at the genus level. Principal coordinate analysis (PCoA) was performed using (g) Euclidean, (h) Abundance‑Jaccard, and (i) Bray‐curtis dissimilarity measures to assess β‑diversity and visualize treatment‑induced shifts in microbial community structure (*n* = 5 for each group, PERMANOVA test). (j–m) Analysis of the relative abundance of (j) *Bacteroides*, (k) *Enterococcus*, (l) *Bifidobacterium* and (m) *Ligilactobacillus* in the periodontal region (*n* = 5 for each group, Wilcoxon rank‐sum test). (n) LEfSe used to display the dominant bacteria in different groups (phylum to genus level) with an LDA value > 2. Diameter and color of each node indicate its enrichment in the corresponding groups. (o) The heatmap illustrates the relative abundance distribution of the 20 top dominant bacteria across different samples (genus level), with the abscissa representing group names and the ordinate representing bacterial taxonomy at the family level. Different color gradients (from blue to red) indicate the magnitude of relative abundance. The clustering dendrograms on the top and left reflect the similarity relationships of abundance patterns. (p) Comparison of the abundance of various microorganisms (*n* = 5 for each group, Wilcoxon rank‐sum test). The data are presented as the mean ± SD of *n* = 5. ns: no significance, ^*^ *p* < 0.05, ^**^ *p* < 0.01, ^***^ *p* < 0.001, ^****^ *p* < 0.0001.

Periodontitis alters the physicochemical and biological properties of the subgingival microenvironment, promoting expansion of Gram‐negative anaerobes at the expense of commensal Gram‐positive bacteria and leading to dysbiosis. Mo_5_N_6_ nanozymes, with POD‐ and OXD‐like activities, efficiently clear pathogens, while CAT‐like activity dynamically regulates oxidative stress and oxygen levels, facilitating microbial homeostasis. To assess the impact of Mo_5_N_6_‐based nanozyme therapy on subgingival microbiota, 16S rDNA sequencing was performed (Figure 5a). Compared with the Control group, the Saline group and the H_2_O_2_ group exhibited a significant reduction in α‐diversity. However, following treatment with Mo_5_N_6_ or Mo_5_N_6_ + H_2_O_2_, these α‐diversity indices moderately increased. The combined treatment group had the highest α‐diversity, suggesting an improvement in subgingival microbial diversity and abundance (Figure 5b–e). Subsequently, principal coordinate analysis (PCoA) showed that the subgingival microbiota of the Saline group clustered distinctly from that of the Control group in multidimensional space, indicating substantial differences in microbial community composition between the two groups. The clustering of the low‐concentration H_2_O_2_ group overlapped extensively with that of the Saline group, suggesting minimal disruption to the community structure by H_2_O_2_ alone. In contrast, moderate separation was observed between the Mo_5_N_6_ group and the Saline group, potentially due to the OXD‐like activity of Mo_5_N_6_ exerting mild inhibitory effects on certain microbial taxa. The AT group also exhibited a closer clustering towards the healthy group, attributed to its antibacterial potential. Notably, the Mo_5_N_6_ + H_2_O_2_ group exhibited a pattern that markedly deviated from the Saline group and was the most closely aligned with the Control group, indicating greater potential of the combination therapy in restoring periodontal microbial ecological homeostasis (Figure 5g–i). Circos plot and Community barplot analysis illustrate the relative abundance of microbial communities at the phylum, class, and genus levels across different treatment groups, providing a visual overview of the dynamic shifts in subgingival microbiota following ligature induction and subsequent interventions. The results indicated that the subgingival microbiota was primarily dominated by *Streptococcus spp*. In the Saline group, the relative abundance of several pathogenic taxa significantly increased, whereas in the treatment groups, pathogenic bacteria were reduced and the relative abundance of beneficial bacteria was notably restored (Figure 5f; Figures S27a,b and S28). Further genus‐level analysis of the average relative abundance of the same species across different groups revealed that, compared to the healthy controls, the periodontitis mice exhibited significant enrichment of potential or opportunistic pathogens, including *Bacteroides, Enterococcus, Proteus, Acinetobacter, Salmonella, and Pseudomonas* (Figure 5j–m,p). These taxa represent key drivers of microbial dysbiosis and impaired oral health, closely associated with inflammation‐induced tissue destruction. However, the relative abundance of these genera markedly decreased following treatment with Mo_5_N_6_ + H_2_O_2_. In contrast, beneficial genera such as *Bifidobacterium* and *Ligilactobacillus* were significantly enriched after treatment. This may be due to the fact that, after ROS‐mediated clearance of the microbiota, the CAT‐like activity of Mo_5_N_6_ generates O_2_, which further inhibits the recolonization of Gram‐negative bacteria [57]. Previous studies have reported that probiotic formulations containing *Bifidobacterium* and *Ligilactobacillus* exert positive effects on oral microbiota health and macrophage repolarization, which plays a pivotal role in inflammation resolution and periodontal tissue recovery [58, 59]. Clinically, adjunctive probiotic therapy has been shown to significantly improve key periodontal parameters, including bleeding index, probing depth, and clinical attachment level [60, 61]. Consistent with these reports, in vitro experiments showed that conditioned supernatants derived from *Bifidobacterium breve* and *Ligilactobacillus salivarius* significantly promoted the repolarization of macrophages pretreated by lipopolysaccharide (LPS) from an inflammatory (M1‐like) state toward a pro‐resolving (M2‐like) phenotype, as evidenced by RT‐qPCR analysis of representative markers (TNF‐α and CD86 for M1, Arg‐1 and IL‐10 for M2) (Figure S29). The increased colonization and dominance of such probiotics observed in the present study may partially account for the therapeutic benefits in periodontitis mice. Further Spearman rank‐based correlation test investigating the correlation between clinical variables, including bone quality parameters and inflammatory factors, and the disease‐associated genera also supported our point. The subgingival microbiome was closely associated with periodontitis status. And the significant differences in microbial composition between the Saline group and the Mo_5_N_6_ + H_2_O_2_ therapy group might be a key reason for the improvement in periodontitis (Figure S30).

LEfSe (Linear Discriminant Analysis Effect Size), by integrating the Kruskal–Wallis test with Linear Discriminant Analysis (LDA), enables the identification of significantly different taxa among sample groups across multiple taxonomic levels, from order to genus. In this study, the phylogenetic cladogram and LDA bar chart generated by LEfSe (Figure 5n; Figures S31 and S32) clearly illustrated shifts in representative microbial taxa among different groups: the periodontitis group was dominated by Gram‐negative bacteria such as *Enterobacteriaceae* and *Bacteroidaceae*, whereas the Mo_5_N_6_ + H_2_O_2_ treatment group exhibited significant enrichment of Gram‐positive bacteria, particularly *Bifidobacteriaceae*. Additionally, the heatmap of microbial communities demonstrated marked differences between the Control and Saline groups, while the Mo_5_N_6_ + H_2_O_2_ group held a distribution trend the closest to the Control among all treatment groups (Figure 5o; Figure S33). This finding is highly consistent with the aforementioned α‐ and β‐ diversity analyses, further underscoring the potential of Mo_5_N_6_ combined with H_2_O_2_ intervention in modulating subgingival microbial structure and promoting the dominance of beneficial bacteria.

In conclusion, our findings reveal that Mo_5_N_6_ + H_2_O_2_ therapy induces a distinct remodeling of the oral microbiota, emphasizing its central role in periodontitis progression and offering a promising avenue for periodontal regeneration through precise modulation of local microbial communities.

### 2.5 Biocompatibility Evaluation of Mo_5_N_6_

Finally, we systematically evaluated the biosafety of Mo_5_N_6_, including cytotoxicity assays, hemolysis testing, and in vivo toxicity assessment. In the cytotoxicity assay, the CCK‐8 method and Live/Dead staining were used to evaluate the effect of Mo_5_N_6_ and Mo_5_N_6_ + H_2_O_2_ on mouse L929 cells and human gingival fibroblasts, with H_2_O_2_‐only groups at multiple concentrations in comparison. A slight decrease in cell viability was observed only at high concentrations of Mo_5_N_6_ or Mo_5_N_6_ + H_2_O_2_ (Figures S34 and S35). Similarly, the hemolysis assay was employed to assess the potential of Mo_5_N_6_ to disrupt red blood cells, and the results indicated no apparent hemolytic toxicity (Figure S36). Further histological examination of major organs including the heart, liver, spleen, lungs, and kidneys by H&E staining revealed no observable pathological damage or inflammatory infiltration, confirming its in vivo biosafety (Figure S37). Collectively, these results demonstrate the excellent biocompatibility of this nanozyme material and its potential for application in clinical studies of periodontitis.

## 3 Conclusion

In this study, we successfully developed Mo_5_N_6_ nanozymes with multi‐enzyme‐like catalytic activities, enabling efficient synergistic therapy against periodontitis by dynamically modulating the subgingival redox microenvironment. Leveraging Mo‐N coordination centers, Mo_5_N_6_ efficiently catalyzed H_2_O_2_ and O_2_ to generate a localized burst of ROS within the gingival sulcus, enabling effective in situ antibacterial activity and inducing irreversible oxidative damage to ROS‐sensitive periodontal pathogens. Following local administration at inflamed periodontal sites, Mo_5_N_6_ significantly promoted the regeneration of alveolar bone and periodontal tissues, as evidenced by a 250 µm reduction in the CEJ‐ABC distance and a 2.1‐fold increase in bone volume fraction. This therapeutic effect was further enhanced by inflammation resolution through the promotion of macrophage polarization toward the anti‐inflammatory M2 phenotype, ultimately achieving simultaneous regeneration of both bone tissue and collagen fibers. Meanwhile, Mo_5_N_6_ treatment markedly restored the α‐diversity of the subgingival microbiota, and β‐diversity analyses revealed a microbial community shift toward a healthy state, characterized by a notable decrease in pathogenic bacteria and a 3.8‐fold increase in probiotic genera such as *Bifidobacterium* and *Ligilactobacillus*. These changes facilitated the reconstruction of a symbiotic microbiota network with immune regulatory functions. Collectively, this study proposes a cascade therapeutic strategy of “ROS‐mediated pathogen eradication followed by probiotic‐facilitated colonization resistance,” offering a novel microbiota‐directed nanotherapeutic approach for periodontitis and other chronic inflammatory diseases.

## 4 Experimental Section

### 4.1 Reagents and Materials

MoO_3_, 3,3′,5,5′‐Tetramethylbenzidine dihydrochloride hydrate (TMB), Methylene blue (MB), Rhodamine B (RhB), L‐Ascorbic acid (AA), Titanic sulfate and Crystal violet were bought from Macklin Reagent (Shanghai, China). 9,10‐Anthracenediyl‐bis(methylene)dimalonic Acid (ABDA), Antifade mounting medium and Saline were bought from Beyotime Biotechnology (Shanghai, China). Sodium acetate, 5,5‐Dimethyl‐1‐pyrroline N‐oxide (DMPO) were purchased from Aladdin Bio‐Chem Tech‐nology Co (Shanghai). Brain heart infusion (BHI) broth was purchased from Oxoid Limited (UK). Hemin chloride, EDTA decalcification solution, vitamin K, artificial saliva, 4% paraformaldehyde and glacial acetic acid were purchased from Solarbio (Beijing, China). All chemical reagents were obtained from commercial companies and used as applicable without further purification.

### 4.2 Synthesis of Mo_5_N_6_

Typically, the molybdenum precursor was placed in a ceramic boat and heated to 900°C at a rate of 5 °C/min under an Ar/ NH_3_ mixed gas atmosphere for 480 min. The resulting black product was collected and stored for later use.

### 4.3 Characterization of Mo_5_N_6_

The crystal structure of the resultant products was characterized by X‐ray diffraction (XRD) using an X‘PertProMPD (Holand) D/max‐γAX‐ray diffractometer with Cu Kα radiation (λ = 0.154 178 nm). Scanning electron microscopy (SEM) images and energy dispersive X‐ray (EDS) spectroscopy were performed by a SU8100 scanning electron microscope with an acceleration voltage of 20 kV. Transmission electron microscopy (TEM) and high‐resolution TEM (HRTEM) images were obtained with an FEI‐Tecnai F20 (200 kV), respectively. The TEM samples were prepared by dropping the solution onto a copper grid with a polyvinyl supporting film and dried in air. Raman measurements were carried out using the LabRam HR Evolution system using the 532 nm line of an argon ion laser as the excitation source. The Fourier transform infrared (FTIR) spectrum is recorded on an FTIR spectrometer (Spectrum One, PerkinElmer) using a standard KBr pellet technique. X‐ray photoelectron spectroscopy (XPS) was obtained by using a KRATOS Axis ultra‐DLD X‐ray photoelectron spectrometer with a Mo_5_N_6_ ochromatized Mg Kα X‐ray source (hν = 1283.3 eV).

### 4.4 Peroxidase‐Like Activity of Mo_5_N_6_

The POD‐like activity of Mo_5_N_6_ was determined based on the colorimetric reaction of TMB in the presence of H_2_O_2_. Briefly, Mo_5_N_6_ (100 µg mL^−1^), TMB (0.4 mM) and H_2_O_2_ (200 µM) were added to the HAc‐NaAc buffer (pH 4). Hitachi UH4150 UV–vis spectrophotometer was used to record the absorbance of the color reactions after a certain reaction time, and the chromogenic reactions represent POD‐like activity. The initial reaction temperature of the reaction system was controlled to 37°C.

### 4.5 The Determination of Catalytic Kinetics

The steady‐state kinetic assays were performed at 37 °C in a buffer solution (1 mL, pH 4) with Mo_5_N_6_ (100 µg mL^−1^) in various tubes. Then, TMB solution (20 µL, 9.6 mg mL^−1^ in DMSO) and various H_2_O_2_ concentrations (final concentrations of 0.01, 0.05, 0.1, 0.2, 0.4, and 0.8 mM) were added to the above mixture, followed by measuring the absorbance at 652 nm, and the Michaelis‐Menten constant was determined based on the Michaelis‐Menten saturation curve. Kinetic parameters to evaluate enzymatic properties were calculated using the Lineweaver‐Burk plot.

$$\;\frac{1}{V_{0}}=\frac{K_{m}}{V_{max}\left[s\right]}+\frac{1}{V_{max}}$$

where *V*_0_ is the initial reaction velocity. *K*_m_ is the apparent Michaelis‐Menten constant. *V*_max_ is the maximum initial velocity. [*s*] represents the substrate concentration.

### 4.6 Detection of Hydroxyl Radical (·OH)

The generation of ·OH was evaluated by an EPR spectroscopy spectrometer using a DMPO spin‐rapping adduct. During the experiment, the concentration of Mo_5_N_6_, DMPO, and H_2_O_2_ were 100 µg mL^−1^, 50 mM and 200 µM, respectively. All mixtures were dispersed in pH 4 HAc‐NaAc buffer. The solutions were then aspirated into quartz capillaries for EPR analysis.

### 4.7 Evaluation of ·OH Generation by MB/RhB Decoloration

Mo_5_N_6_ nanozymes (300 µL, final concentration of 100 µg mL^−1^) were added in MB/RhB solution (3 mL, 10 µg mL^−1^) containing H_2_O_2_ (10 mM). After incubation for different time intervals (0, 2, 4, 6, 8, 10, 20, and 30 min), the MB/RhB aqueous solution was centrifuged to remove the Mo_5_N_6_ nanozymes. The absorbance was measured by using a UV‐Vis spectrophotometer.

### 4.8 Computational Method

All density functional theory (DFT) calculations were carried out using the Vienna Ab initio Simulation Package (VASP) within the generalized gradient approximation (GGA) [62, 63], employing the Perdew‐Burke‐Ernzerhof (PBE) exchange‐correlation functional [64]. The interactions between valence electrons and ionic cores were described using the projector augmented‐wave (PAW) method [65, 66], and the electronic wavefunctions were expanded in a plane‐wave basis set with a kinetic energy cutoff of 450 eV. The pristine bulk structure of Mo_5_N_6_ was fully optimized prior to surface construction, using a Monkhorst‐Pack k‐point mesh of 5 × 5 × 2. Van der Waals interactions were taken into account via the DFT‐D3 empirical correction scheme. Structural relaxations were performed until the residual forces on each atom were below 0.02 eV Å^−1^, with the total energy convergence criterion set to 1 × 10^−5^ eV. For surface calculations, a 3 × 3 × 1 Monkhorst‐Pack k‐point grid was employed. Spin polarization was explicitly included in all calculations. To mimic bulk constraints, atoms in the bottom half of the slab were fixed during geometry optimization. The lattice parameters used in the simulations were a = 10.58 Å, b = 9.85 Å, c = 24.42 Å, with α = 90°, β = 90°, and γ = 90.15°. After geometry optimization, the projected density of states (PDOS) was calculated using a denser 5 × 5 × 1 Monkhorst‐Pack k‐point mesh to ensure sufficient resolution of the electronic structure.

### 4.9 Bacterial Culture and Preparation

Wide‐type Chl^r^ *E. coli* (ATCC 25 922) and *MRSA* (ATCC 22 004) were used as the models of Gram‐negative and Gram‐positive bacterium strains, respectively. The bacteria grown on Luria‐Bertani (LB) agar plate were transferred to 50 mL of LB at 37°C and shaken for 24 h, respectively. Bacteria were harvested by centrifuging (7200 rpm for 1 min) and then washed with deionized water three times. The supernatant was discarded and the remaining bacteria were resuspended in deionized water to a concentration of ∼2 × 10^8^ Colony‐Forming Units (CFU) mL^−1^. *Porphyromonas gingivalis* (W83) and *Fusobacterium nucleatum* (ATCC 25 586), two representative periodontal pathogens, were cultured under anaerobic conditions (5% H_2_, 15% CO_2_, 80% N_2_, 37°C). Frozen bacterial stocks were inoculated onto BHI blood agar plates supplemented with hemin chloride and vitamin K, and incubated in an anaerobic chamber at 37°C. Single colonies were then transferred to 5 mL of BHI broth supplemented with hemin chloride and vitamin K for expansion. After 2 days of incubation, the cultures were centrifuged at 7200 rpm for 1 min to collect the bacteria, which were then washed, resuspended, and adjusted to a final concentration of approximately 2 × 10^8^ CFU mL^−1^. *Bifidobacterium breve* (CMCC 1.3001) and *Ligilactobacillus salivarius* (BNCC 194 719) were utilized as probiotic models in this study. The frozen bacterial stocks were revitalized by streaking onto De Man, Rogosa and Sharpe (MRS) agar plates and incubated at 37 °C in an anaerobic chamber (5% H_2_, 15% CO_2_, 80% N_2_, 37 °C) for 24 h. The culture medium for *B. breve* was supplemented with 0.05% (w/v) L‐cysteine hydrochloride.

### 4.10 Cell Culture and Preparation

The human gingival fibroblasts used in this study were obtained from YuchiCell (Shanghai, China). They were cultured at 37°C in a humidified atmosphere with 5% CO_2_ using α‐Minimum Essential Medium (α‐MEM) (Gibco, USA) supplemented with 10% fetal bovine serum (FBS, Gibco, USA) and 1% penicillin‐streptomycin solution (P/S, Gibco, USA). L929 cells and RAW264.7 macrophages were cultured at 37°C in a humidified atmosphere with 5% CO_2_ using Dulbecco's modified Eagle's medium (DMEM, high glucose) (Gibco, USA) containing 10% FBS and 1% P/S.

### 4.11 Antibacterial Activity In Vitro

Representative pathogenic bacteria including Chl^r^ *E. coli*, *MRSA*, Pg and Fn were selected to investigate the bacterial capture and eradication abilities of the Mo_5_N_6_ nanozyme catalytic activity. The bacteriostatic effect was evaluated by agar plate counting. A 10 mL bacterial suspension (approximately 10^4^ CFU mL^−1^) containing H_2_O_2_ (final concentration 200 µM) and Mo_5_N_6_ nanozymes (concentration 100 µg mL^−1^) was co‐incubated for 2 h, after which colony counting was performed to obtain the final bacterial quantity. The bactericidal performance and bacterial capture ability of these systems were visualized by SEM. After treatment, the bacterial suspensions were fixed with 2.5 wt.% glutaraldehyde and dehydrated through a graded ethanol/water series. SEM images were then acquired to observe bacterial capture and morphological changes.

### 4.12 Bacterial Protein Leakage Measurement

The Pg suspension was treated with H_2_O_2_ and/or Mo_5_N_6_ for 2 h and then centrifuged at 5000 rpm for 10 min at 4°C to collect the supernatant. Subsequently, the supernatant was detected by a bicinchoninic acid (BCA) protein assay from Beyotime (Shanghai, China) to assess the amount of protein leakage.

### 4.13 Bacterial Nucleic Acid Leakage Measurement

The supernatant was prepared as described above and the absorbance at λ = 260 nm was measured, corresponding to the amount of nucleic acid released.

### 4.14 Bacteria Membrane Permeability

The membrane permeability of Pg was evaluated by an ortho‐nitrophenyl‐β‐galactoside (ONPG) hydrolysis assay. The Pg biofilm was treated with H_2_O_2_ and/or Mo_5_N_6_ for 48 h and then tested through an ONPG assay from Solarbio (Beijing, China). The OD_405_ values of the supernatant were measured.

### 4.15 Crystal Violet Biofilm Staining

96‐well plates were coated with artificial saliva overnight. Subsequently, 100 µL of logarithmic‐phase Pg or Fn bacterial suspensions (10^8^ CFU mL^−1^) were inoculated into each well. The plates were anaerobically incubated at 37°C for 96 h to allow biofilm formation, with an additional 100 µL of BHI broth supplemented at 48 h. Following biofilm maturation, 100 µL of different treatment materials were added to the wells and incubated for another 48 h. After treatment, the supernatant was discarded, and wells were gently washed with PBS. Biofilms were fixed with 4% paraformaldehyde for 10 min, then the fixative was removed. Each well was stained with 200 µL of 0.1% (w/v) crystal violet solution for 30 min. Excess dye was removed by washing three times with PBS. Finally, biofilms were solubilized by adding 30% glacial acetic acid and thoroughly shaken on a shaker. The absorbance of the resulting solution was measured at 570 nm using a microplate reader (Biotek, ELX808, USA). The biofilm biomass of each sample was calculated using the following formula:

$$\mathrm{Biofilm}\;\mathrm{formation}(\%)=\frac{OD_{570\mathrm{Exp}}-OD_{570\mathrm{Blank}}}{OD_{570\mathrm{Control}}-OD_{570\mathrm{Blank}}}\times 100\%\;$$

The absorbance values of the experimental group, negative control, and blank control were recorded as OD_570Exp_, OD_570Control_ and OD_570Blank_, respectively.

### 4.16 Live/Dead Staining

Glass coverslips were placed in 48‐well plates. Bacterial attachment and subsequent material treatments were performed as described above. After discarding the supernatant, the wells were gently washed with PBS to remove non‐adherent bacteria. The remaining surface‐associated bacteria were then stained using a Live & Dead Bacterial Staining Kit (Beyotime, Shanghai, China) for 45 min in the dark. After sealing with antifade mounting medium, samples were observed using confocal laser scanning microscopy (CLSM) (STELLARIS 8, Leica, Germany).

### 4.17 Establishment and Treatment of the Mouse Periodontitis Model

Male C57BL/6 mice (6–8 weeks, 18–22 g body weight) were purchased from Vital River Corp (Beijing, China). The animals were kept in an environment complying with the NIH guidelines for the care and use of laboratory animals. All animal experiments were conducted using protocols approved by the Institutional Animal Care and Use Committee at the Institute of Tumors at the Chinese Academy of Medical Sciences (NCNST21‐202503‐0021).

Referring to previous studies, an experimental periodontitis model in mice was established using the ligature‐induced method (*n* = 8). After anesthesia, mice in the periodontitis group and all treatment groups received ligatures around the maxillary second molars using sterile 5‐0 silk sutures and minimally invasive instruments. The condition of the ligatures was checked daily for loosening or the need for adjustment. Subsequently, different treatments were administered to each group with a micro‐injection system and a 34‐gauge needle by injecting 10 µL of the corresponding materials into four sites of gingival culcus around the second molar: the buccal center, lingual center, mesial, and distal. Proficiency in the injection technique was ensured to prevent iatrogenic injury to the gingival tissues. Two hours after material application, gingival crevicular plaque samples were collected for bacterial colony counting. After 7 days of treatment, the mice were euthanized via cervical dislocation, and maxillary alveolar bone samples were harvested and fixed in 4% paraformaldehyde.

### 4.18 Micro‐CT Analysis

The collected maxillary bone samples were scanned using micro‐CT (SkyScan 1276, Bruker, Belgium) with parameters set at 50 kV, 200 µA, and a resolution of 6 µm. Three‐dimensional reconstruction was performed using CTvox software, while two‐dimensional reconstruction and quantitative analysis were carried out using DataViewer and CTAn software. The alveolar bone surrounding the second molar and the interradicular region were selected as regions of interest (ROI). Parameters such as the distance from the cementoenamel junction to the alveolar bone crest (CEJ‐ABC), bone volume to tissue volume ratio (BV/TV) and trabecular thickness (Tb.Th) were quantified.

### 4.19 Histological Analysis of Periodontal Tissues

The fixed maxillary bone samples were decalcified in EDTA decalcifying solution for 4 weeks, embedded in paraffin and sectioned into 4 µm slices. According to the manufacturers' protocols, sections from each group were subjected to H&E staining (Hematoxylin‐Eosin (HE) Stain Kit, Solarbio, China), Masson's trichrome staining (Masson's Trichrome Stain Kit, Solarbio, China), and tartrate‐resistant acid phosphatase (TRAP) staining (Tartrate‐Resistant Acid Phosphatase (TRAP) Stain Kit, Solarbio, China). Osteoclasts were identified based on TRAP‐positive signals and the presence of three or more nuclei, and counted independently by three blinded investigators. Images were captured using a fluorescence microscope (Olympus, Tokyo, Japan).

### 4.20 Immunofluorescence Staining of Periodontal Tissue

The tissue sections were sequentially processed through the following steps: deparaffinization, rehydration, antigen retrieval, permeabilization, and blocking. Subsequently, they were incubated overnight with the following primary antibodies: anti‐F4/80 (dilution 1:50, sc‐377009, Santa Cruz), anti‐iNOS (dilution 1:100, ab178945, Abcam), anti‐CD206 (dilution 1:100, ab64693, Abcam), anti‐IL‐1β (dilution 1:100, BF8021, Affinity), anti‐TNF‐α (dilution 1:100, ab307164, Abcam), or anti‐IL‐10 (dilution 1:100, 60 269, Proteintech). Subsequently, the sections were incubated with corresponding fluorochrome‐conjugated secondary antibodies (Alexa Fluor Plus 488 and Alexa Fluor Plus 647, Invitrogen). Nuclei were counterstained using an anti‐fade mounting medium containing DAPI (Servicebio, China). The stained sections were visualized under a confocal laser scanning microscope (CLSM), and the fluorescence intensity of the markers was quantified using ImageJ software.

### 4.21 In Vivo Antibacterial Assay

Two hours after subgingival injection of the materials, microbial plaque samples were collected from the gingival sulcus of mice in each group using sterile swabs. The swabs were immediately immersed in saline and shaken at 200 rpm for 30 min at room temperature to obtain a bacterial suspension. 100 µL of the diluted solution of suspension was then spread onto BHI blood agar plates. The plates were incubated under standard conditions (5% CO_2_, 37°C) for 48 h, after which the bacterial colonies were counted.

### 4.22 In Vitro Biocompatibility Evaluation

The Cell Counting Kit‐8 (CCK‐8; Dojindo, Kumamoto, Japan) was used according to the manufacturer's instructions. L929 cells and human gingival fibroblasts were seeded in 96‐well plates at a density of 5×10^3^ cells per well. After cell attachment, the culture medium was replaced with medium containing gradient concentrations (0–500 µg mL^−1^) of Mo_5_N_6_, medium containing 100 µg mL^−1^ Mo_5_N_6_ combined with gradient concentrations of H_2_O_2_ or only gradient concentrations of H_2_O_2_ (0–500 µM). And the cells were incubated for 1, 3, and 5 days. The absorbance at 450 nm was measured for the experimental groups (OD_450Exp_), the negative control group (OD_450Control_), and the background group (OD_450Blank_). The cell viability (%) was calculated using the following formula:

$$\mathrm{Cell}\;\mathrm{viability}(\%)=\frac{OD_{450\mathrm{Exp}}-OD_{450\mathrm{Blank}}}{OD_{450\mathrm{Control}}-OD_{450\mathrm{Blank}}}\times 100\%\;$$

For live/dead staining, L929 cells and human gingival fibroblasts were seeded in 24‐well plates at a density of 2×10^4^ cells per well. After attachment, the cells were treated with culture medium containing gradient concentrations (0–500 µg mL^−1^) of Mo_5_N_6_ nanoparticles for 48 h. Cell viability was then assessed using a Calcein‐AM/EthD‐III Live/Dead Cell Double Stain Kit (Solarbio, China). After 15 min of staining, the cells were observed under a fluorescence microscope (Olympus, Tokyo, Japan) to detect green (live) and red (dead) fluorescence.

### 4.23 Hemolysis Assay

Mouse blood samples were washed with saline and centrifuged (1000 rpm, 10 min) to isolate red blood cells (RBCs). Then, 1 mL of Mo_5_N_6_ solutions at gradient concentrations (0–500 µg mL^−1^) was added to the experimental group. An equal volume of ddH_2_O was added to the positive control group, and an equal volume of PBS was added to the negative control group. After mixing, the samples were incubated at 37°C for 6 h, followed by centrifugation and photography. The absorbance of the supernatant at 540 nm was measured for the experimental group (OD_540Exp_), the negative control group (OD_540Control_), and the background group (OD_540Blank_). The hemolysis rate was calculated using the following formula:

$$\mathrm{Hemolysis}\;\mathrm{rate}(\%)=\frac{OD_{540\mathrm{Exp}}-OD_{540\mathrm{Blank}}}{OD_{540\mathrm{Control}}-OD_{540\mathrm{Blank}}}\times 100\%\;$$

### 4.24 In Vivo Biocompatibility Evaluation

Major organs (heart, liver, spleen, lungs, and kidneys) collected from mice in each group were subjected to histological analysis. Paraffin sections were prepared and stained with H&E following the aforementioned procedures. The stained sections were imaged using a fluorescence microscope (Olympus, Tokyo, Japan).

### 4.25 16S rDNA Analysis of the Periodontal Subgingival Microbiome

After treatment, the ligature wires were collected from each group and immediately stored at −80°C. Among these, the ligature wires from the Control group were ligated and then retrieved immediately to serve as a baseline reference representing the normal periodontal microbiome. Total genomic DNA of the microbial community was extracted from ligature wires using the E.Z.N.A. soil DNA Kit (Omega Bio‐tek, Norcross, GA, U.S.) according to the manufacturer's instructions. The concentration and purity of the DNA were assessed using a NanoDrop2000 spectrophotometer (Thermo Scientific, USA). Quantitative PCR (qPCR) was performed using PowerUp SYBR Green Master Mix (Thermo Fisher Scientific, USA). The hypervariable V3‐V4 region of the bacterial 16S rRNA gene was amplified with the barcoded forward primer 338F (5′‐ACTCCTACGGGAGGCAGCAG‐3′) and the reverse primer 806R (5′‐GGACTACHVGGGTWTCTAAT‐3′) on an ABI GeneAmp 9700 PCR thermocycler. The PCR products were separated on a 2% agarose gel, purified using the PCR Clean‐Up Kit (YuHua, Shanghai, China), and quantified with a Qubit 4.0 fluorometer (Thermo Fisher Scientific, USA). Sequencing libraries were constructed with the NEXTFLEX Rapid DNA‐Seq Kit and paired‐end sequencing was performed on an Illumina Nextseq2000 platform (Majorbio Bio‐Pharm Technology Co., Ltd., Shanghai, China). The raw sequences were quality‐filtered using fastp (version 0.19.6) and merged with FLASH (version 1.2.11). Denoising was performed using the DADA2 plugin within the QIIME2 (version 2020.2) pipeline to obtain amplicon sequence variants (ASVs). All sequences annotated as chloroplasts or mitochondria were removed. To minimize the effects of uneven sequencing depth, the number of sequences per sample was rarefied to 20 000 for subsequent analysis. Taxonomic assignment of ASVs was performed using the Naive Bayes classifier in QIIME2 against the SILVA 16S rRNA database (v138). Phylogenetic Investigation of Communities by Reconstruction of Unobserved States (PICRUSt2, version 2.2.0) was employed to predict metagenomic functions. All bioinformatic analyses were conducted on the Majorbio Cloud Platform (https://cloud.majorbio.com).

### 4.26 In Vitro Macrophage Treatment and Polarization

RAW264.7 macrophages were seeded into 12‐well plates and allowed to adhere overnight prior to stimulation. To induce an inflammatory macrophage phenotype, RAW264.7 cells were pretreated for 12 h with 100 ng mL^−1^ lipopolysaccharide derived from Pg (Sigma‐Aldrich, USA). *Bifidobacterium breve* and *Ligilactobacillus salivarius* were cultured for 24 h, and supernatants were collected by centrifugation and sterile filtration (0.22 µm) and then applied to inflammatory macrophages for an additional 12 h. Cells were then harvested for reverse transcription quantitative RT‐qPCR analysis of related genes (TNF‐α, CD86, IL‐10, and Arg‐1). Pg was cultured for 48 h and treated with H_2_O_2_ and/or Mo_5_N_6_ for 2 h, followed by centrifugation to collect the bacterial pellet, which was then resuspended and further cultured for 24 h. The resulting culture supernatant was collected, sterile‐filtered (0.22 µm), and diluted to treat RAW264.7 macrophages for 12 h. Subsequently, total RNA was extracted for RT‐qPCR analysis of TNF‐α, CD86, IL‐10, and Arg‐1 expression.

### 4.27 RNA Extraction and RT‐qPCR

Total RNA was extracted using TRIzol reagent and reverse‐transcribed into cDNA using PrimeScript RT Mix (Takara Bio, Japan) following standard protocols. RT‐qPCR was performed using PowerUp SYBR Green Master Mix (Thermo Scientific, USA). Gene expression levels were normalized to β‐actin, and relative expression was calculated using the 2‐ΔΔCt method. The primer sequences utilized for amplifying each gene are listed in Table S3.

### 4.28 Statistical Analysis

Data are presented as mean ± SD. Statistical analyses were performed using one‐way ANOVA followed by Tukey's multiple comparisons test with standard statistical software. The sample size (n) for each experiment is indicated in the corresponding figure legends. Statistical significance was defined as ^*^ *p* < 0.05, ^**^ *p* < 0.01, ^***^ *p* < 0.001, and ^****^ *p* < 0.0001.

## Conflicts of Interest

The authors declare no conflicts of interest.

## Supporting information




**Supporting File**: advs75166‐sup‐0001‐SuppMat.docx.

## Data Availability

The data that support the findings of this study are available from the corresponding author upon reasonable request.
