# Supplementary material for: Multi‐Enzyme Mimetic Molybdenum Nitride Nanozymes Reshape Subgingival Microenvironment for Synergistic Periodontitis Therapy via ROS Regulation and Microbiome Remodeling
Source: Adv Sci (Weinh). 2026 Apr 9;13(41):e17770. doi: 10.1002/advs.202517770 (PMC13326037; doi:10.1002/advs.202517770)
Supplement: Supplementary file 1 — Supporting File: advs75166‐sup‐0001‐SuppMat.docx. [file ADVS-13-e17770-s001.docx]

**Multi-Enzyme Mimetic Molybdenum Nitride Nanozymes Reshape Subgingival Microenvironment for Synergistic Periodontitis Therapy via ROS Regulation and Microbiome Remodeling**

*Weiyu Zhang,^#^ Zhongwei Yang,^#^ Yunfan Zhang,* Longwei Wang, Xiaoyu Zhang, Jiahui Mao, Yizhi Dai, Yifan Yuan, Mingge Wang, Xin Yang, Xin Yu,* Jing Liu,** *Chunying Chen**

W. Zhang and Prof. Y. Zhang

National Center for Stomatology & National Clinical Research Center for Oral Diseases & National Engineering Research Center of Oral Biomaterials and Digital Medical Devices & Beijing Key Laboratory of Digital Stomatology & NHC Key Laboratory of Digital Stomatology & NMPA Key Laboratory for Dental Materials, Beijing 100081, China

W. Zhang

Department of Orthodontics, Peking University School and Hospital of Stomatology, Beijing 100081, China

Y. Zhang

Department of Geriatric Dentistry, Peking University School and Hospital of Stomatology, Beijing 100081, China

Z. Yang, L. Wang, X. Zhang, J. Mao, Y. Dai, Y. Yuan, M. Wang, X. Yang, Prof. J. Liu and Prof. C.Y. Chen

CAS Key Laboratory for Biomedical Effects of Nanomaterials and Nanosafety and CAS Center for Excellence in Nanoscience, National Center for Nanoscience and Technology of China, Beijing 100190, P. R. China

Z. Yang, X. Zhang, X. Yang

Key Laboratory of Resource Biology and Biotechnology in Western China, Ministry of Education, College of Life Sciences, Northwest University, Xi'an, 710069, P. R. China

Prof. X. Yu

Institute for Advanced Interdisciplinary Research (iAIR), School of Chemistry and Chemical Engineering, University of Jinan, Jinan 250022, P. R. China

***Corresponding Authors:**

E-mail: YunfanZhang@bjmu.edu.cn (Yunfan Zhang), ifc_yux@ujn.edu.cn (Xin Yu), jliu@nanoctr.cn (Jing Liu), chenchy@nanoctr.cn (Chunying Chen)

#These authors contributed equally to this work.

Supporting Information


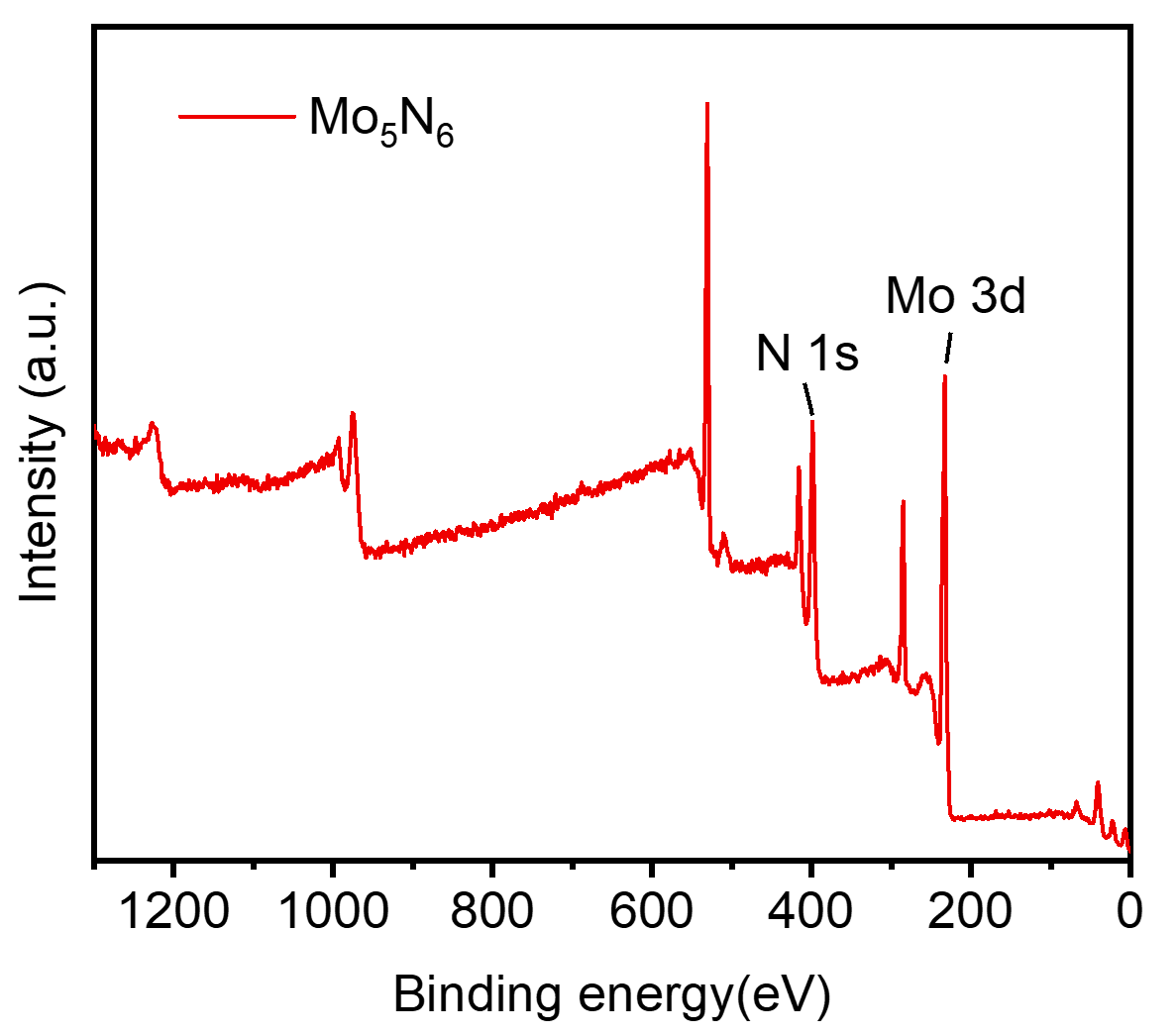


**Figure S1.** The XPS survey spectrum of Mo_5_N_6_.


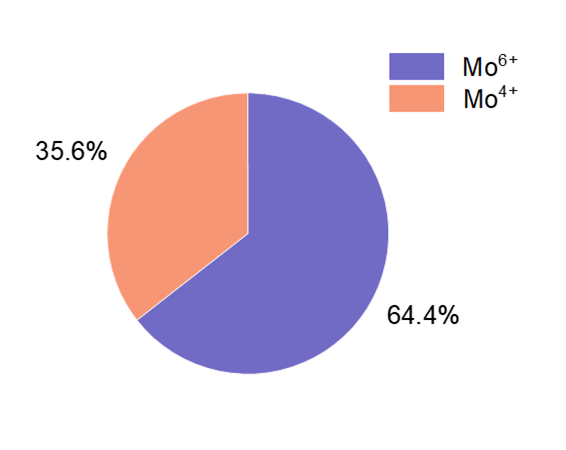


**Figure S2.** Relative proportion of Mo oxidation states derived from XPS peak area analysis.


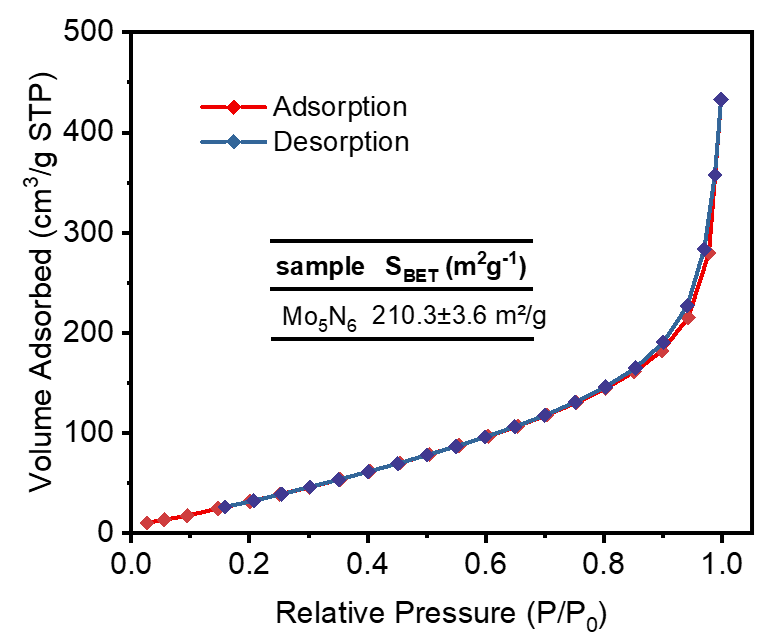


**Figure S3.** Nitrogen adsorption-desorption isotherms. N₂ adsorption at 77 K after degassing at 120 °C for 12 h.


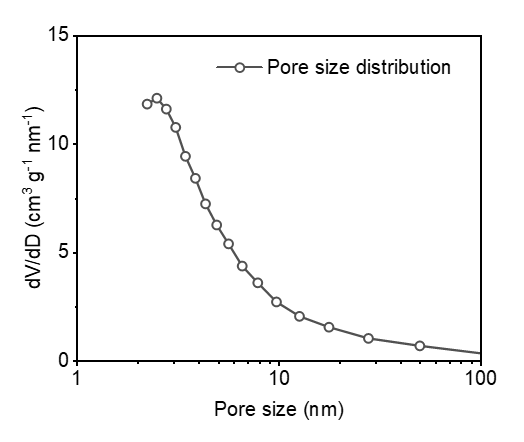


**Figure S4.** Pore size distribution of Mo_5_N_6_.


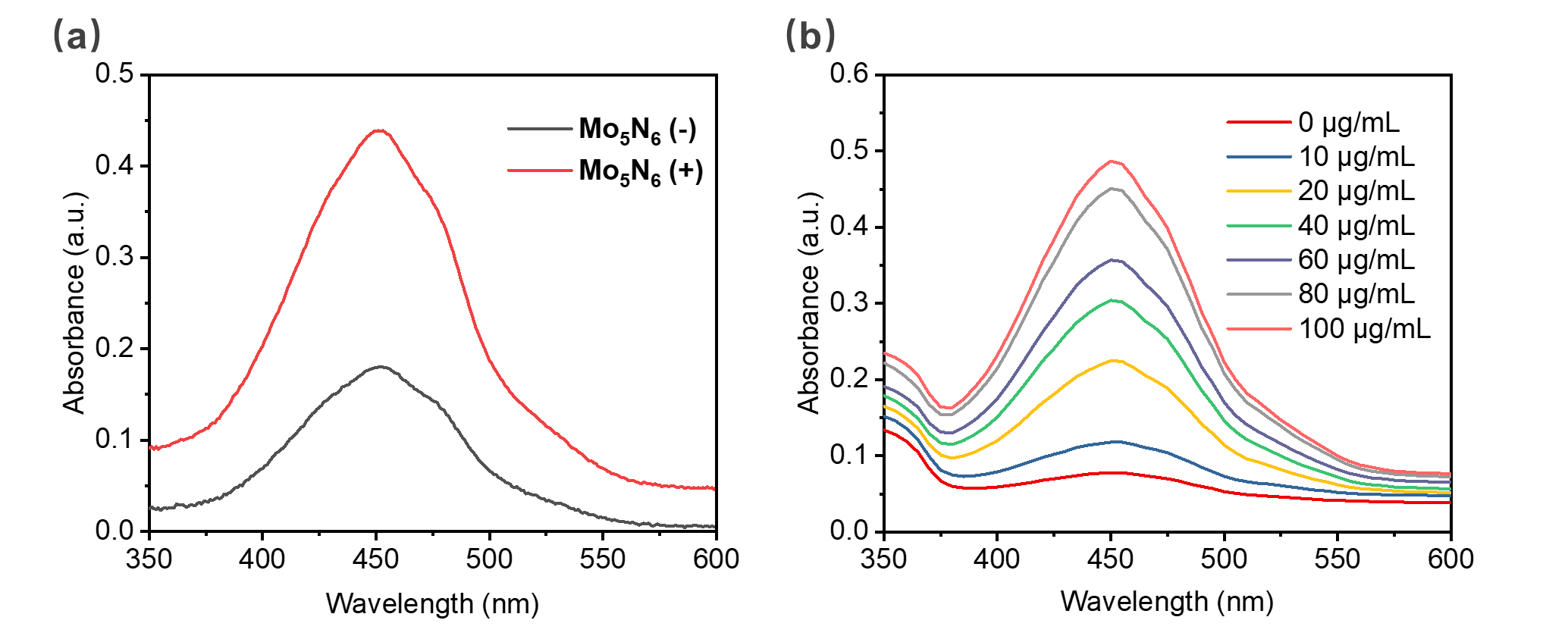


**Figure S5.** Full-spectrum UV-vis absorption profiles of OPD following (a) 100 μg mL^-1^ Mo_5_N_6_ treatment and (b) under incubation with varying concentrations of the material.


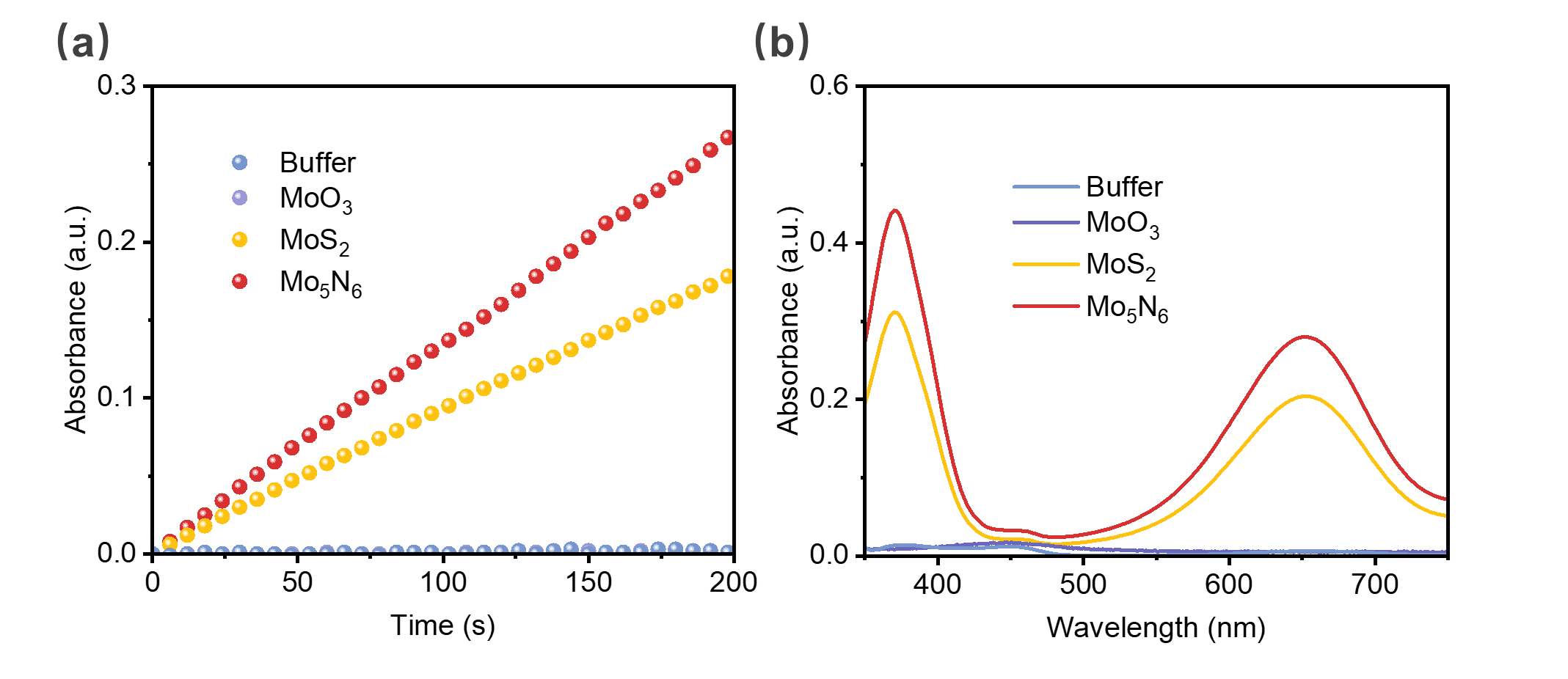


**Figure S6.** (a) Time-dependent changes in absorbance at 652 nm reflecting POD-like activity of various Mo-based materials. (b) UV-vis absorption spectra at the reaction endpoint after co-incubation with different Mo-based materials. The concentration of Mo-based materials (MoO_3_, MoS_2_, and Mo_5_N_6_) was fixed at 100 μg mL^-1^ for all experiments.


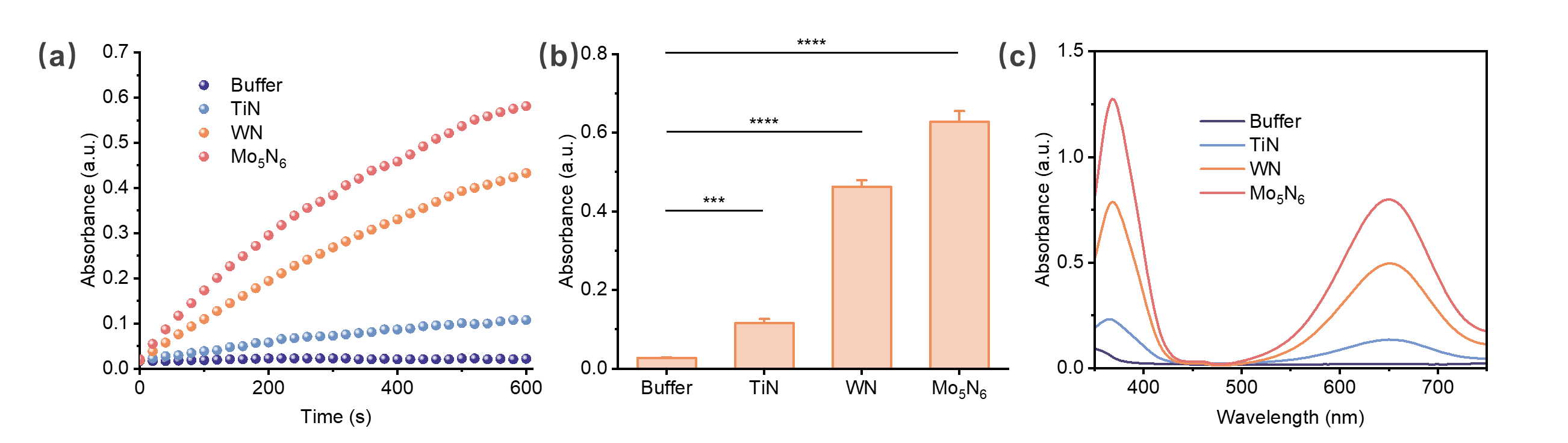


**Figure S7.** POD-like activity of different materials with TMB-H_2_O_2_ substrate: (a) Kinetic curve at 652 nm, (b) Absorbance comparison at 652 nm after reaction, and (c) Full-spectrum absorbance comparison. The concentration of N-based materials (TiN, WN, and Mo_5_N_6_) was fixed at 100 μg mL^-1^ for all experiments. ns: no significance, **p* < 0.05, ***p* < 0.01, ****p* < 0.001, *****p* < 0.0001.


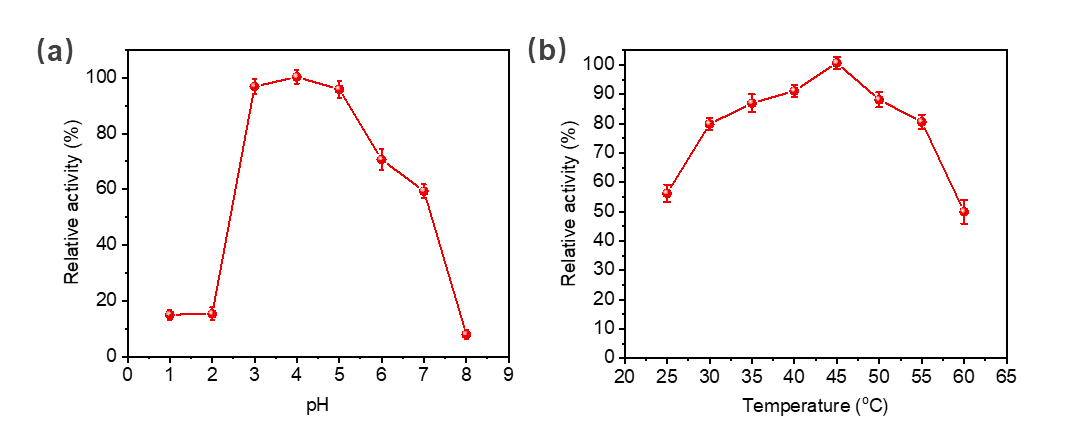


**Figure S8.** Relative peroxidase-like catalytic activity of 100 μg mL^-1^ Mo_5_N_6_ at a) different pH and b) different temperatures. The data are presented as the mean ± standard deviation (SD) of n = 3.


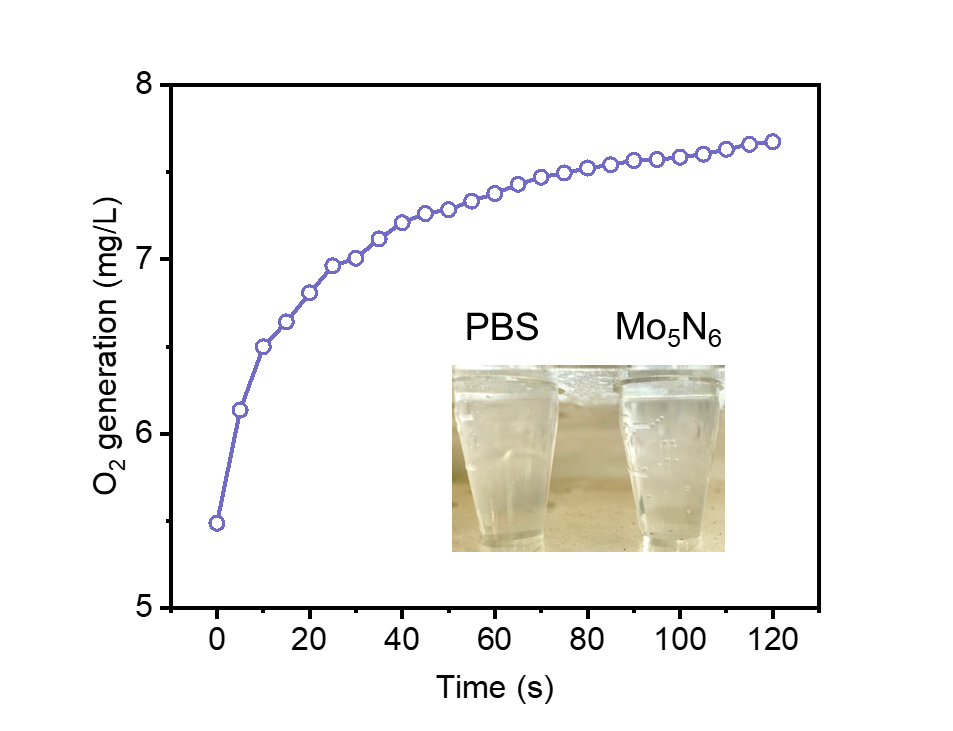


**Figure S9.** Time-dependent O_2_ generation during H_2_O_2_ decomposition with 100 μg mL^-1^ Mo_5_N_6_ and 1 mM H_2_O_2_.


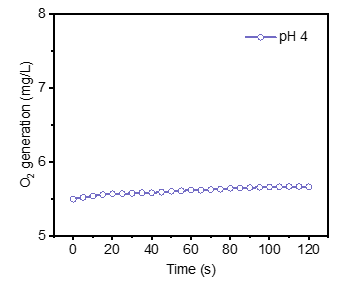


**Figure S10.** Catalytic activity of Mo_5_N_6_ for catalase-like reaction under pH 4 condition. The concentration of Mo_5_N_6_ was fixed at 100 μg mL^-1^ and H_2_O_2_ was fixed at 1 mM.


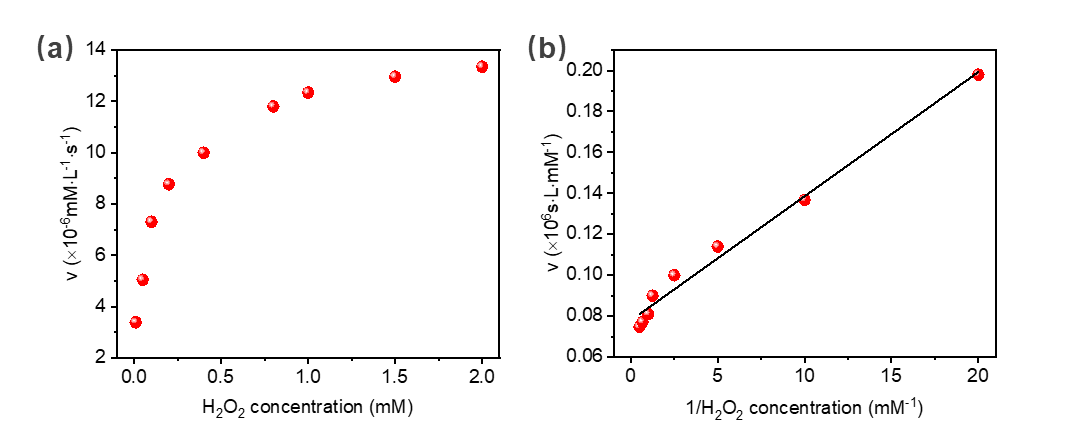


**Figure S11.** Apparent steady-state kinetic study of Mo_5_N_6_ in the presence of H_2_O_2_. (a) Michaelis-Menten kinetic assay and (b) Lineweaver-Burk plotting for Mo_5_N_6_.


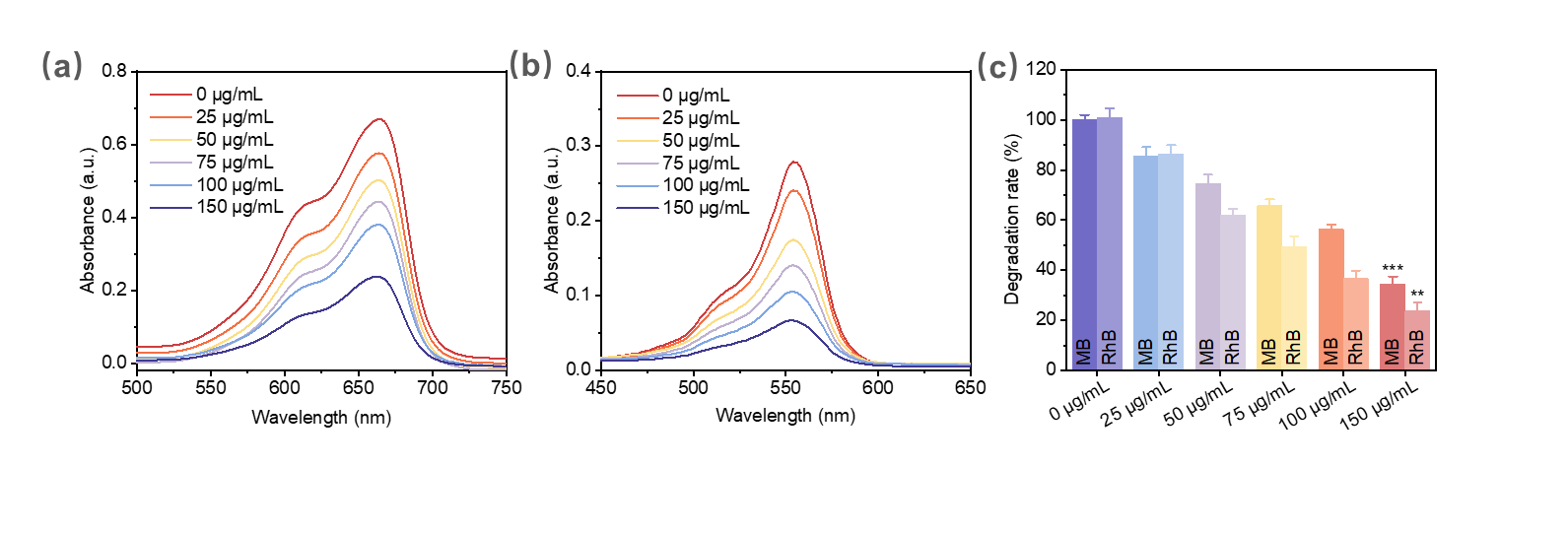


**Figure S12.** UV-vis spectra changes of (a) MB and (b) RhB degradation at graded catalyst loadings. (c) Statistics on degradation efficiencies of MB and RhB (n = 3). The data are presented as the mean ± standard deviation (SD). The significance of the data was calculated by the one-way ANOVA. ns: no significance, **p* < 0.05, ***p* < 0.01, ****p* < 0.001, *****p* < 0.0001.


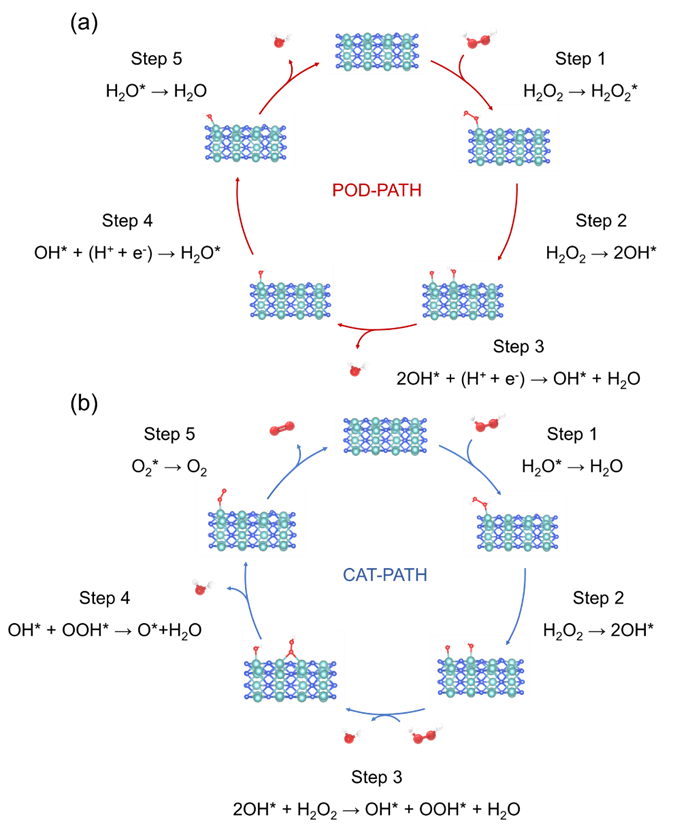


**Figure S13.** Schematic illustration of the POD- and CAT-like reaction pathways for H_2_O_2_ decomposition on the Mo_5_N_6_ surface, showing the adsorption, transformation, and desorption of reaction intermediates.


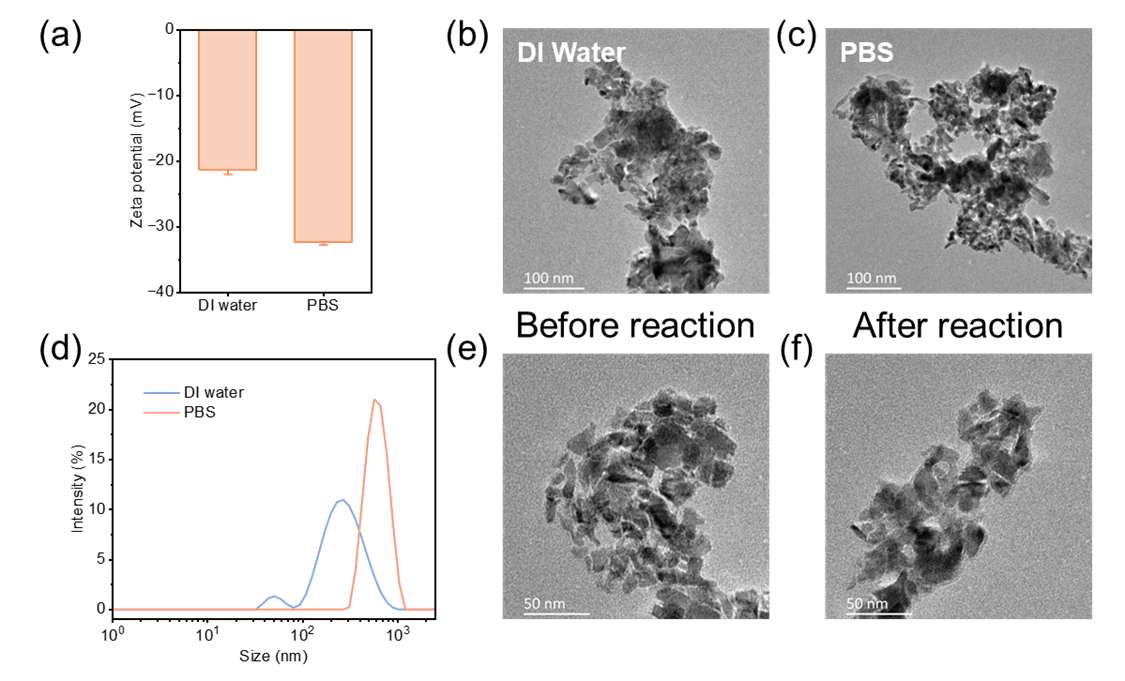


**Figure S14.** Stability of Mo_5_N_6_ (100 μg mL^-1^). (a) Zeta Potential, (b) and (c) TEM characterization of Mo_5_N_6_ after 5-hour soaking in DI water and PBS. (d) Hydrodynamic size distribution of Mo_5_N_6_ measured by dynamic light scattering (DLS) in deionized water (DI water) and PBS. (e) and (f) TEM images of the Mo_5_N_6_ before and after the POD-like catalytic reaction.

**
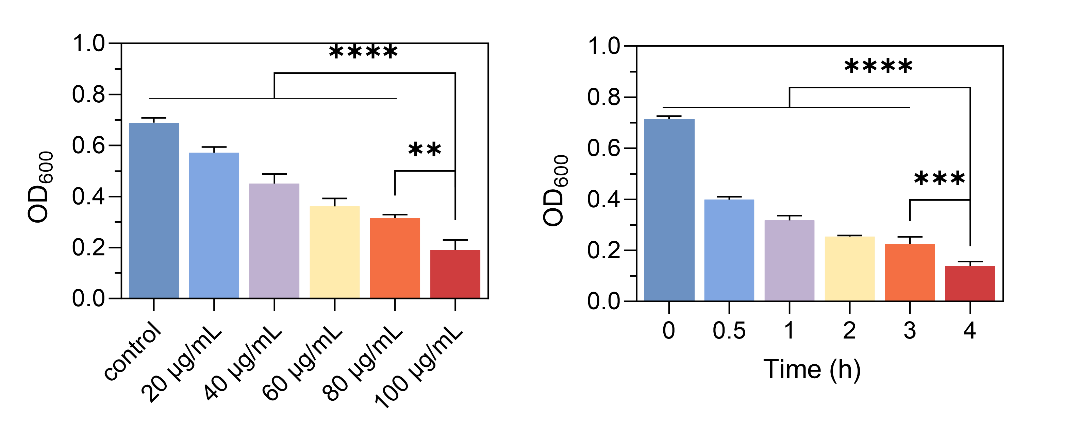
**

**Figure S15.** OD_600_ values of Pg under different (a) Mo_5_N_6_ concentrations (accompanied by 200 μM H_2_O_2_) and (b) incubation times. The data are presented as the mean ± SD of n = 3. The significance of the data was calculated by the one-way ANOVA. ns: no significance, **p* < 0.05, ***p* < 0.01, ****p* < 0.001, *****p* < 0.0001.


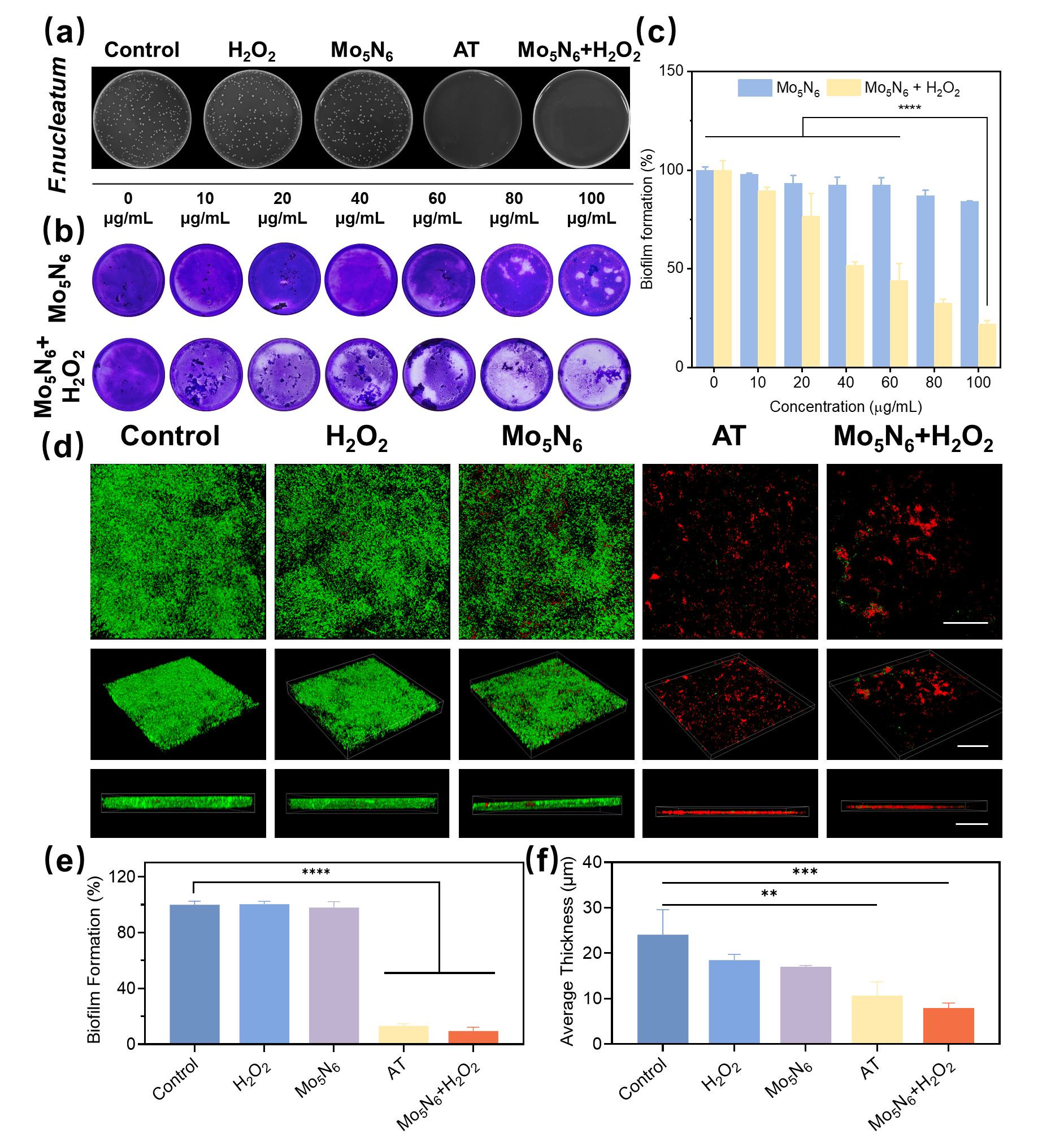


**Figure S16.** *In vitro* antibacterial performance evaluation of Mo_5_N_6_ on Fn. Comparison of Fn across five treatment groups was shown: the untreated control group (Control), the group exposed to 200 µM H_2_O_2_ (H_2_O_2_) or 100 µg mL^-1^ Mo_5_N_6_ alone (Mo_5_N_6_), the group exposed to 3% H_2_O_2_ (active treatment, AT) and the group receiving a combined treatment of 100 µg mL^-1^ Mo_5_N_6_ and 200 µM H_2_O_2_ (Mo_5_N_6_ + H_2_O_2_). (a) Photographs of bacterial colonies formed by Fn. (b) Crystal violet staining images of Fn biofilms under different material concentrations with/without H_2_O_2_ treatment. (c) The biomass of Fn biofilms was quantified by crystal violet staining at OD_595_. (d) 3D CLSM Live/Dead staining images of Fn biofilms after different treatments (scale bar: 50 μm). Statistical analysis of (e) biofilm biomass and (f) average thickness in Panel (d). The data are presented as the mean ± SD of n = 3. The significance of the data was calculated by the one-way ANOVA. ns: no significance, **p* < 0.05, ***p* < 0.01, ****p* < 0.001, *****p* < 0.0001.


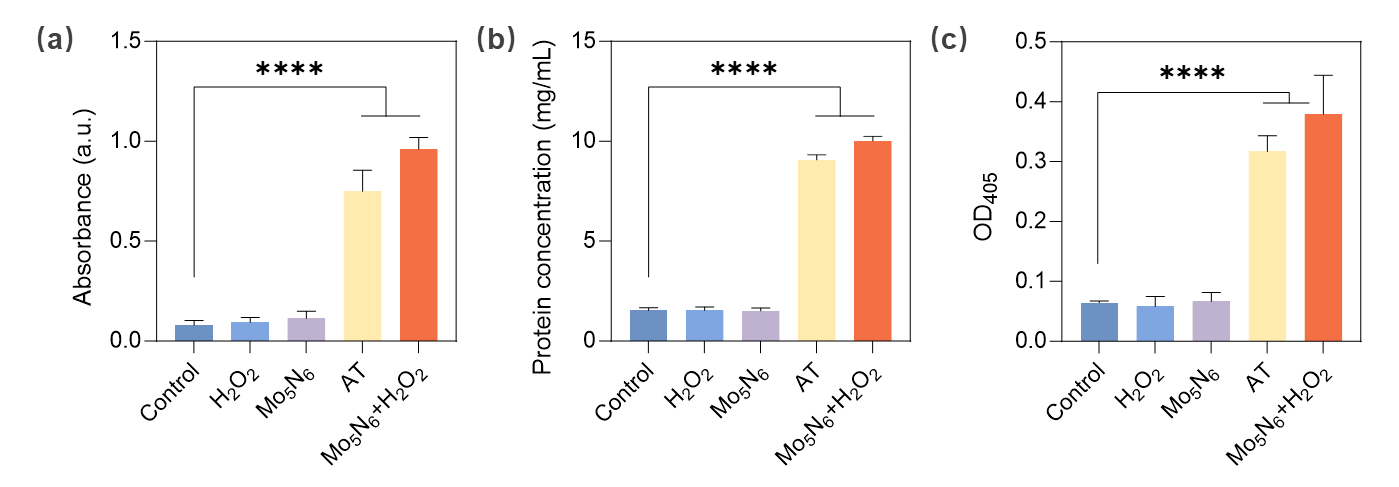


**Figure S17.** Permeability of Pg membrane treated with Mo_5_N_6_ in the absence and presence of H_2_O_2_. (a) OD_260_ values of Pg suspensions treated with Mo_5_N_6_ in the absence and presence of H_2_O_2_. (b) Protein leakage of Pg suspensions treated with Mo_5_N_6_ in the absence and presence of H_2_O_2_ determined by BCA assay. (c) Permeability of Pg membrane determined by ONPG assay. The data are presented as the mean ± SD of n = 3. The significance of the data was calculated by the one-way ANOVA. ns: no significance, **p* < 0.05, ***p* < 0.01, ****p* < 0.001, *****p* < 0.0001.


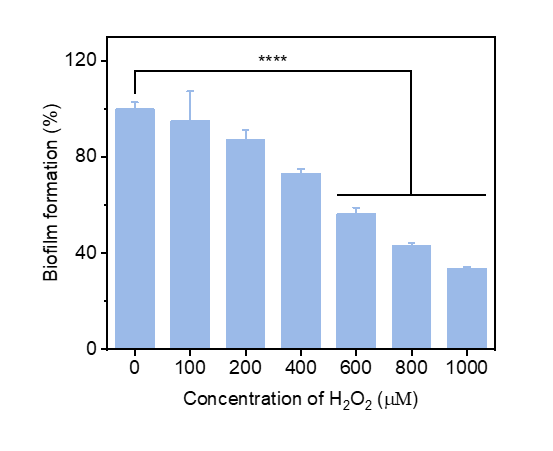


**Figure S18.** The biomass of biofilm stained with crystal violet after treated by different concentrations of H_2_O_2_. The data are presented as the mean ± SD of n = 3. The significance of the data was calculated by the one-way ANOVA. ns: no significance, **p* < 0.05, ***p* < 0.01, ****p* < 0.001, *****p* < 0.0001.


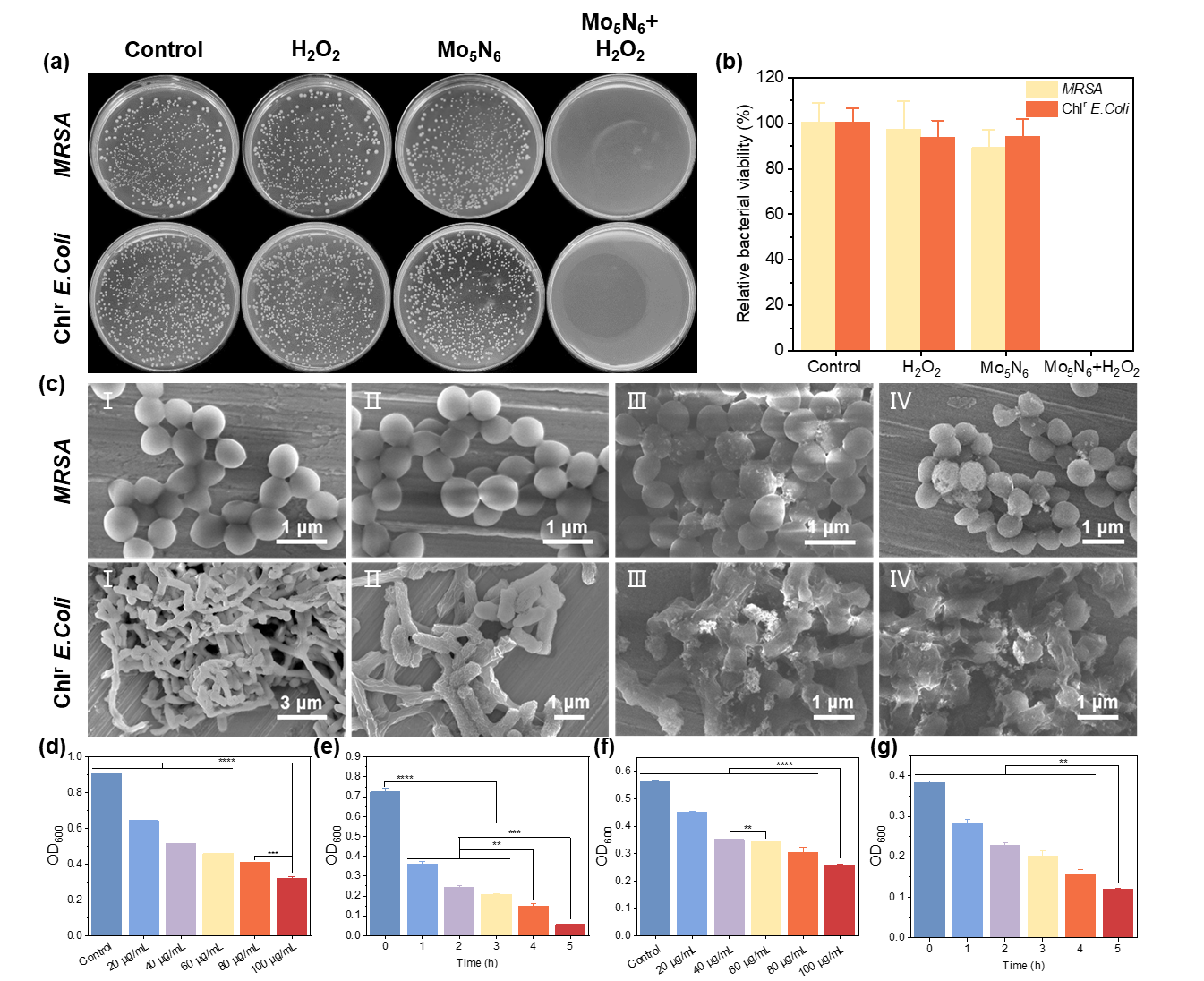


**Figure S19.** *In vitro* antibacterial performance evaluation of Mo_5_N_6_ on *MRSA* and Chl^r^ *E.Coli*. Comparison of *MRSA* and Chl^r^ *E.Coli* across four treatment groups was shown: the untreated control group (Control), the group exposed to 200 µM H_2_O_2_ (H_2_O_2_) or 100 µg mL^-1^ Mo_5_N_6_ alone (Mo_5_N_6_) and the group receiving a combined treatment of 100 µg mL^-1^ Mo_5_N_6_ and 200 µM H_2_O_2_ (Mo_5_N_6_ + H_2_O_2_). (a) Representative images of bacterial colonies cultured from gingival crevicular fluid under various treatments. (b) Relative bacteria viabilities of *MRSA* and Chl^r^ *E.Coli* determined by plate count method. (c) SEM images of *MRSA* and Chl^r^ *E.Coli*. OD_600_ values of (d and e) *MRSA* and (f and g) *E. coli* under different Mo_5_N_6_ concentrations and incubation times. The data are presented as the mean ± SD of n = 3. The significance of the data was calculated by the one-way ANOVA. ns: no significance, **p* < 0.05, ***p* < 0.01, ****p* < 0.001, *****p* < 0.0001.


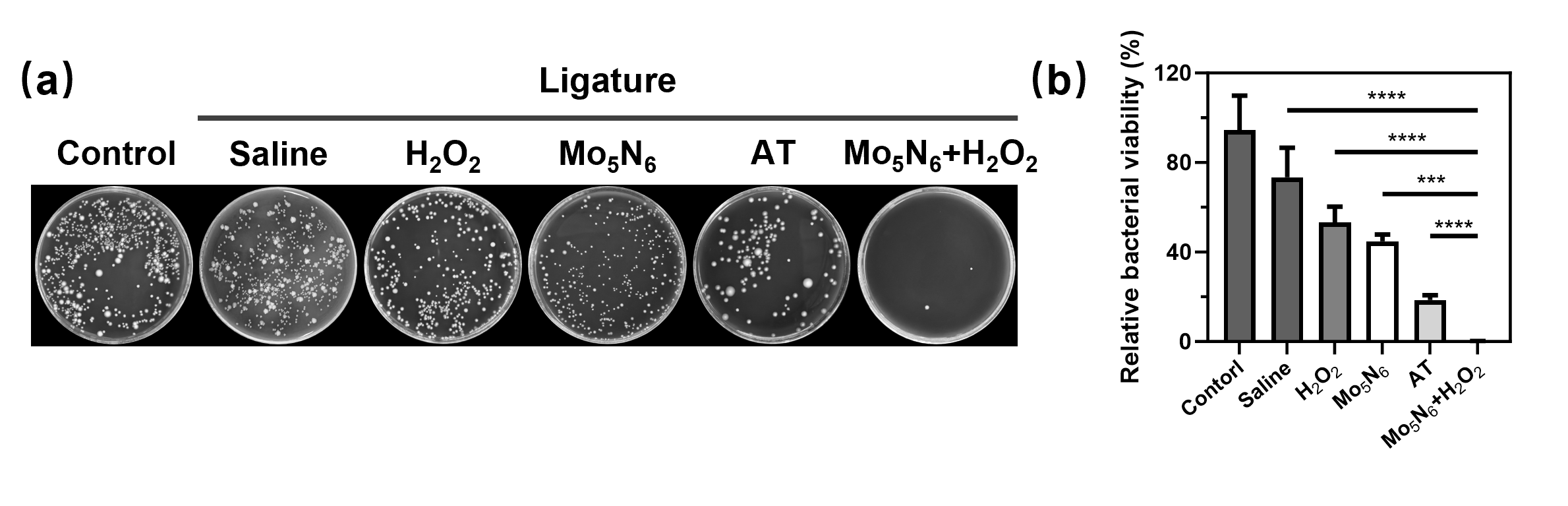


**Figure S20.** *In vivo* antibacterial performance evaluation of Mo_5_N_6_ in mouse ligature-induced periodontitis model. (a) Representative images of bacterial colonies cultured from gingival crevicular fluid. (b) Quantification of bacterial colonies formed from gingival crevicular fluid under different treatment conditions (n = 3). The data are presented as the mean ± SD. The significance of the data was calculated by the one-way ANOVA. ns: no significance, **p* < 0.05, ***p* < 0.01, ****p* < 0.001, *****p* < 0.0001.

**Figure S21.** Quantitative analysis of the trabecular thickness (Tb.Th), representing the average thickness of trabeculae in the alveolar bone (n = 8). The data are presented as the mean ± SD. The significance of the data was calculated by the one-way ANOVA. ns: no significance, **p* < 0.05, ***p* < 0.01, ****p* < 0.001, *****p* < 0.0001.


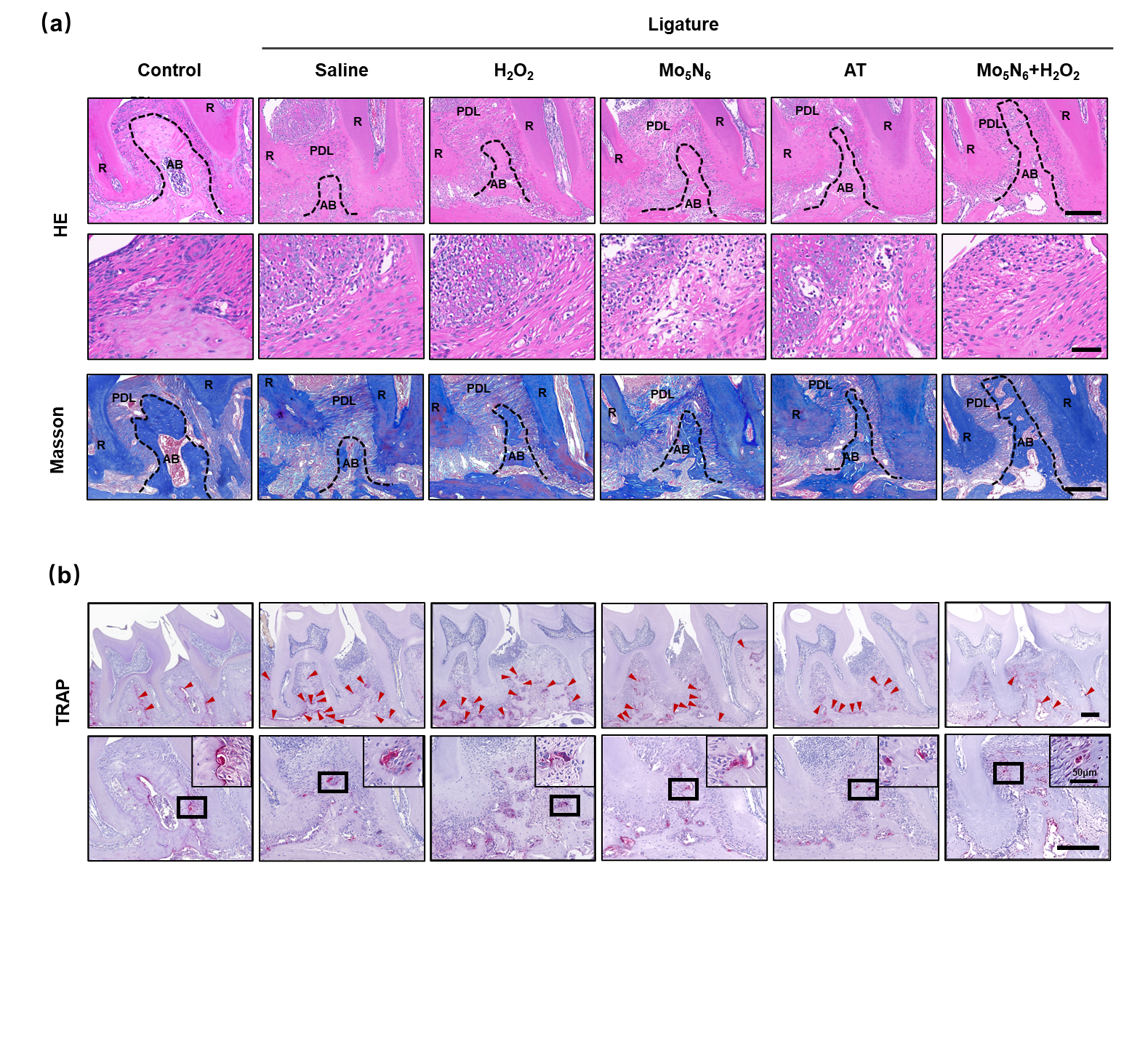


**Figure S22.** Mo_5_N_6_ reduced the periodontal destruction in mouse ligature-induced periodontitis. (a) H&E and Masson staining images of the periodontal tissue (scale bars: 200 μm (first and third rows) and 50 μm (second row), respectively). R, root. PDL, periodontal ligament. AB, alveolar bone. (b) TRAP staining of osteoclasts (red arrowheads indicate osteoclasts that are dyed red) in murine periodontal tissue (scale bar: 200 μm).

**Figure S23.** Semi-quantitative analysis of osteoclasts by TRAP staining. The data are presented as the mean ± SD of n = 3. The significance of the data was calculated by the one-way ANOVA. ns: no significance, **p* < 0.05, ***p* < 0.01, ****p* < 0.001, *****p* < 0.0001.


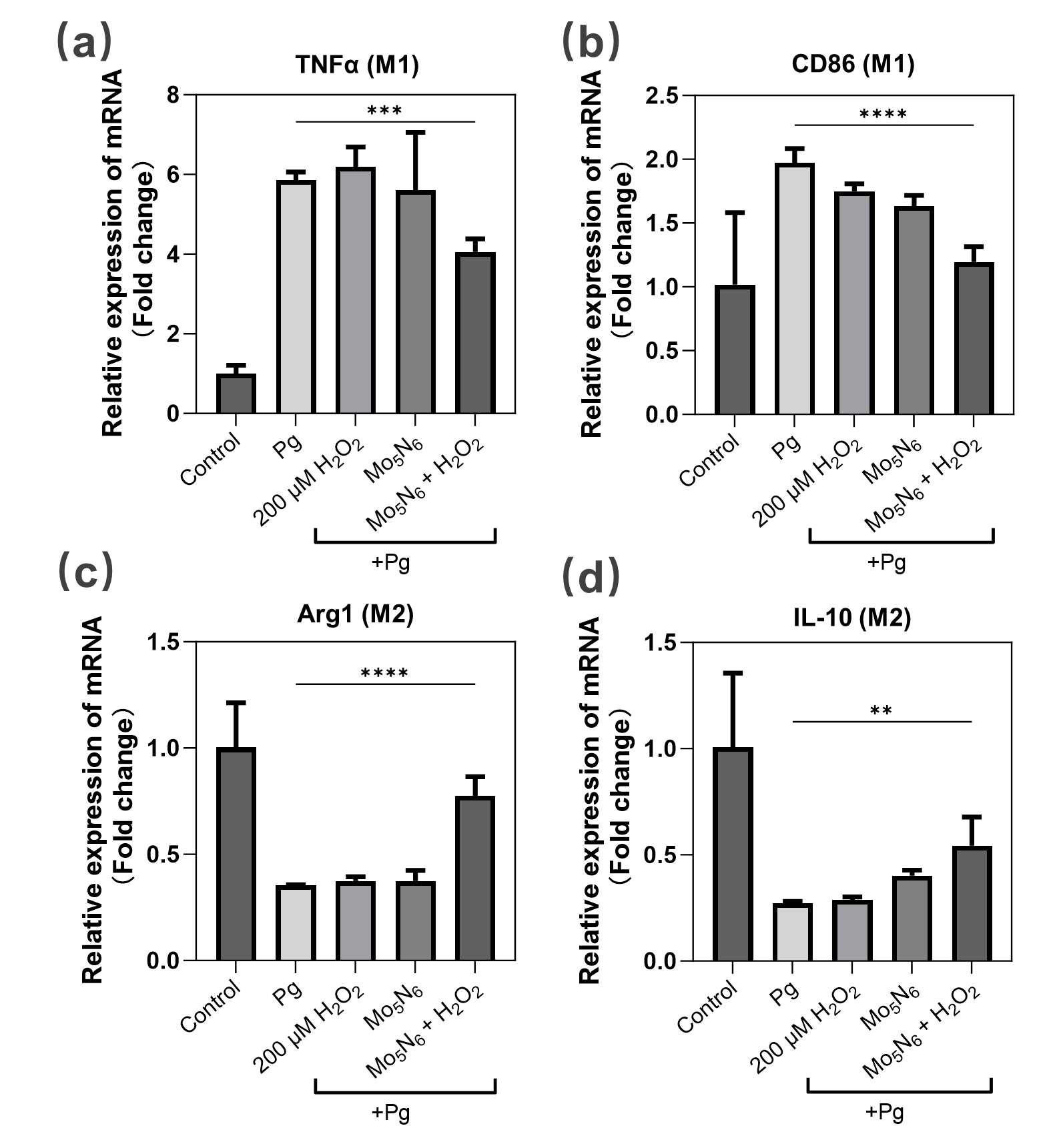


**Figure S24.** RT-qPCR analysis of inflammatory cytokine expression and macrophage polarization. Quantitative analysis of the relative mRNA expression levels for (a) TNF-α, (b) CD86, (c) Arg-1, and (d) IL-10 in RAW 264.7 macrophages. Cells were stimulated with supernatants collected from Pg treated by respective groups (100 μg mL^-1^ Mo_5_N_6_ and/or 200 μM H_2_O_2_). The data are presented as the mean ± SD of n = 3. The significance of the data was calculated by the one-way ANOVA. ns: no significance, **p* < 0.05, ***p* < 0.01, ****p* < 0.001, *****p* < 0.0001.


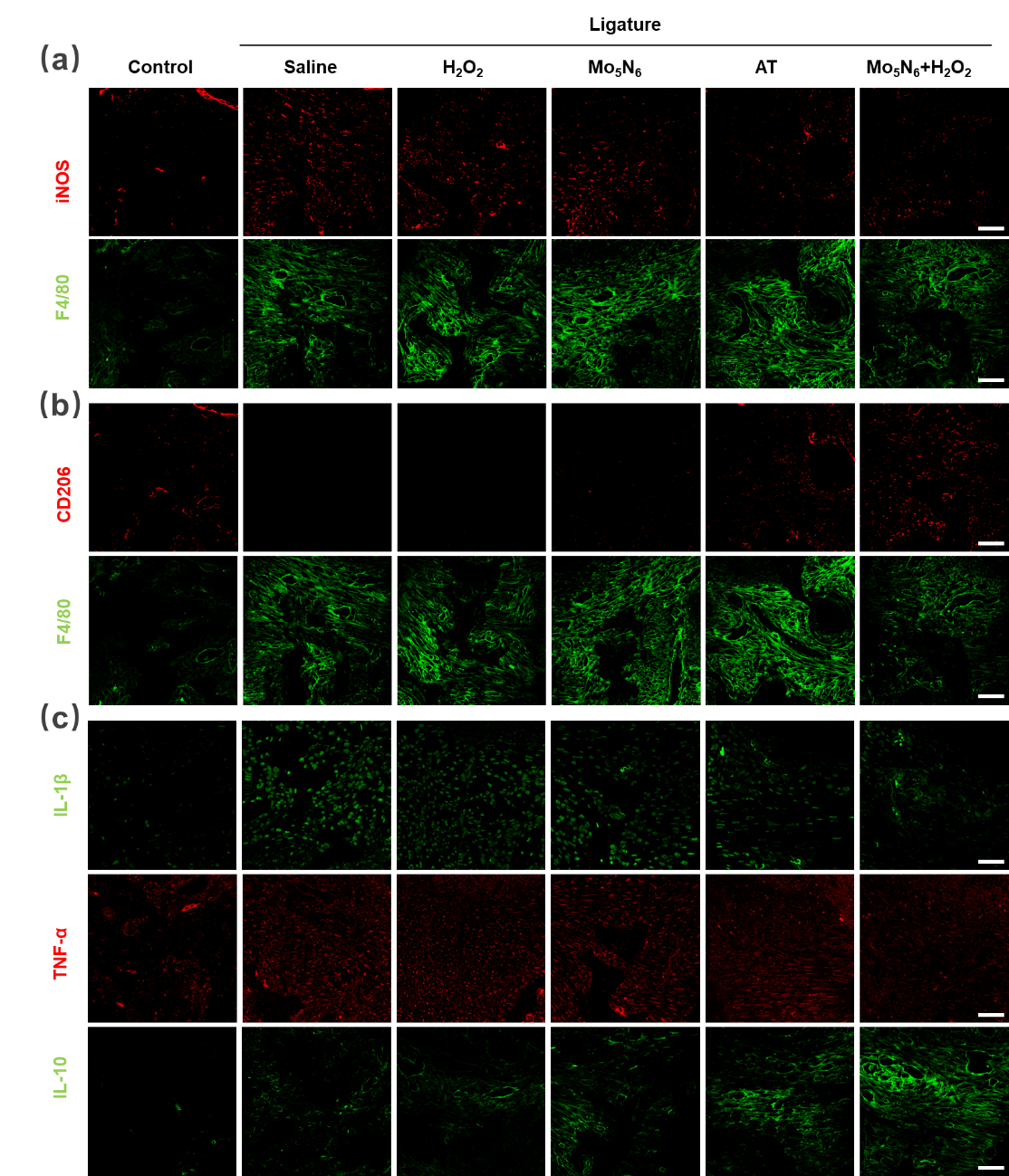


**Figure S25.** Single-channel views from the representative immunofluorescence images of periodontal tissues stained for macrophage polarization and inflammatory factors. (a) Single-channel views showing the expression and localization of iNOS (red) and F4/80 corresponding to the merged image in Figure 4f. (b) Single-channel views showing the expression and localization of CD206 (red) and F4/80 corresponding to the merged image in Figure 4f. (c) Single-channel views showing the expression and localization of IL-1β, TNF-α, and IL-10 corresponding to the merged image in Figure 4g. scale bar: 50 μm, n = 3.


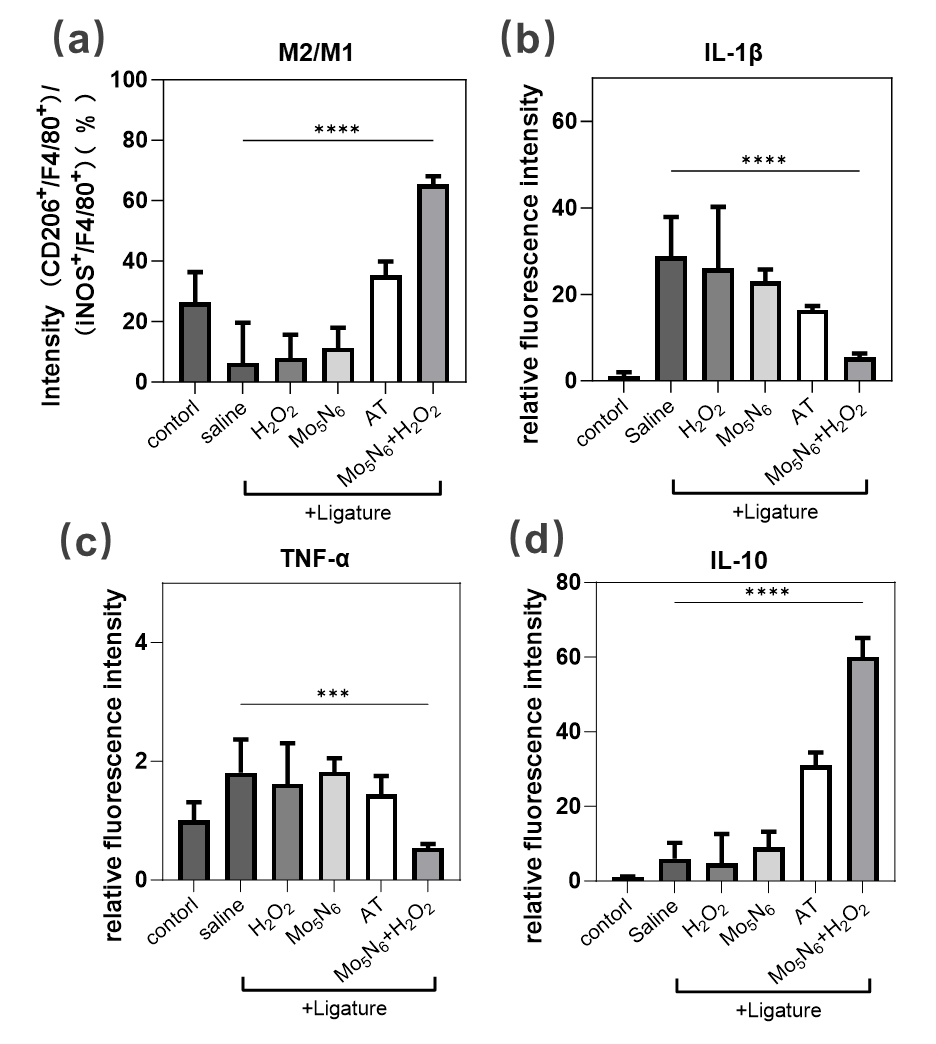


**Figure S26.** Semi-quantitative analysis of immunofluorescence images of periodontal tissues stained for macrophage polarization and inflammatory factors. (a) Quantitative analysis of the macrophage polarization ratio. Quantitative analysis of the relative fluorescence intensity for pro-inflammatory factors (b) IL-1β, (c) TNF-α, and (d) IL-10. The data are presented as the mean ± SD of n = 3. The significance of the data was calculated by the one-way ANOVA. ns: no significance, **p* < 0.05, ***p* < 0.01, ****p* < 0.001, *****p* < 0.0001.


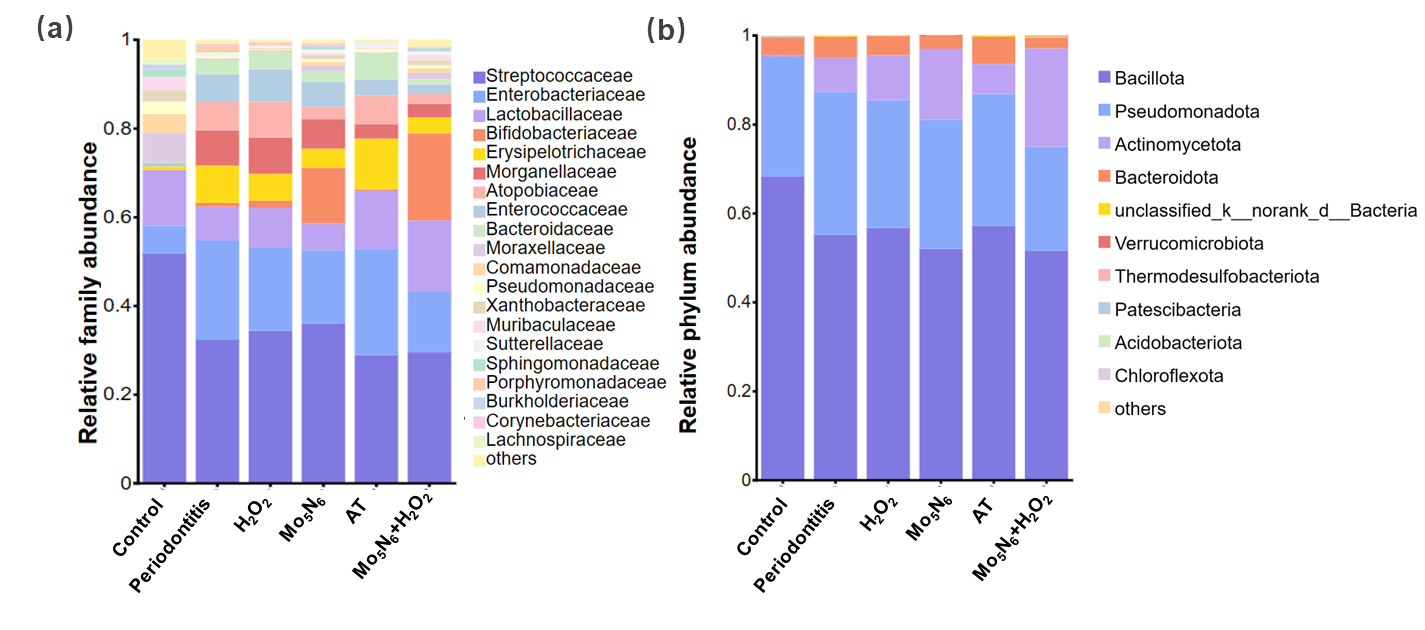


**Figure S27.** Analyses of subgingival microbiome among the control group (Control), ligature + saline group (Saline), ligature + 200 µM H_2_O_2_ group (H_2_O_2_), ligature + 100 µg mL^-1^ Mo_5_N_6_ group, ligature + 3% H_2_O_2_ group (active treatment, AT), and ligature + 100 µg mL^-1^ Mo_5_N_6_ and 200 µM H_2_O_2_ group (Mo_5_N_6_ + H_2_O_2_). (a) Stacked boxplots of the relative abundance of subgingival microbial communities of each group at the family level. (b) Stacked boxplots of the relative abundance of subgingival microbial communities of each group at the phylum level.


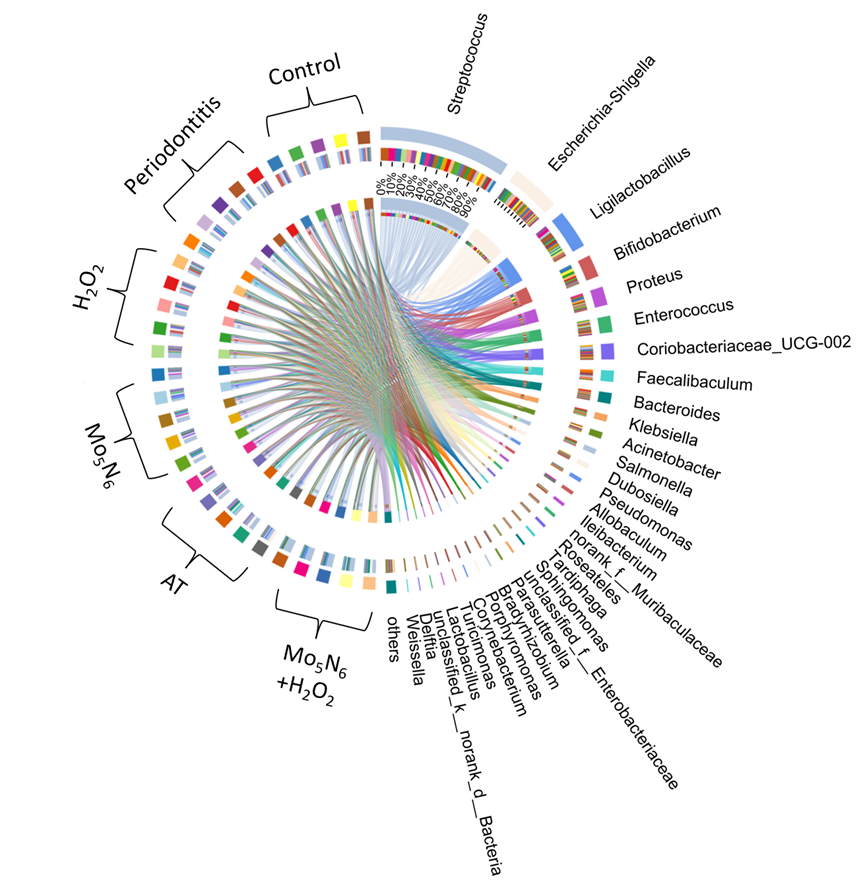


**Figure S28.** Circos plots for sample-species relationships at the genus level. The abundance distribution of different species is illustrated through the connection of inner ribbons.


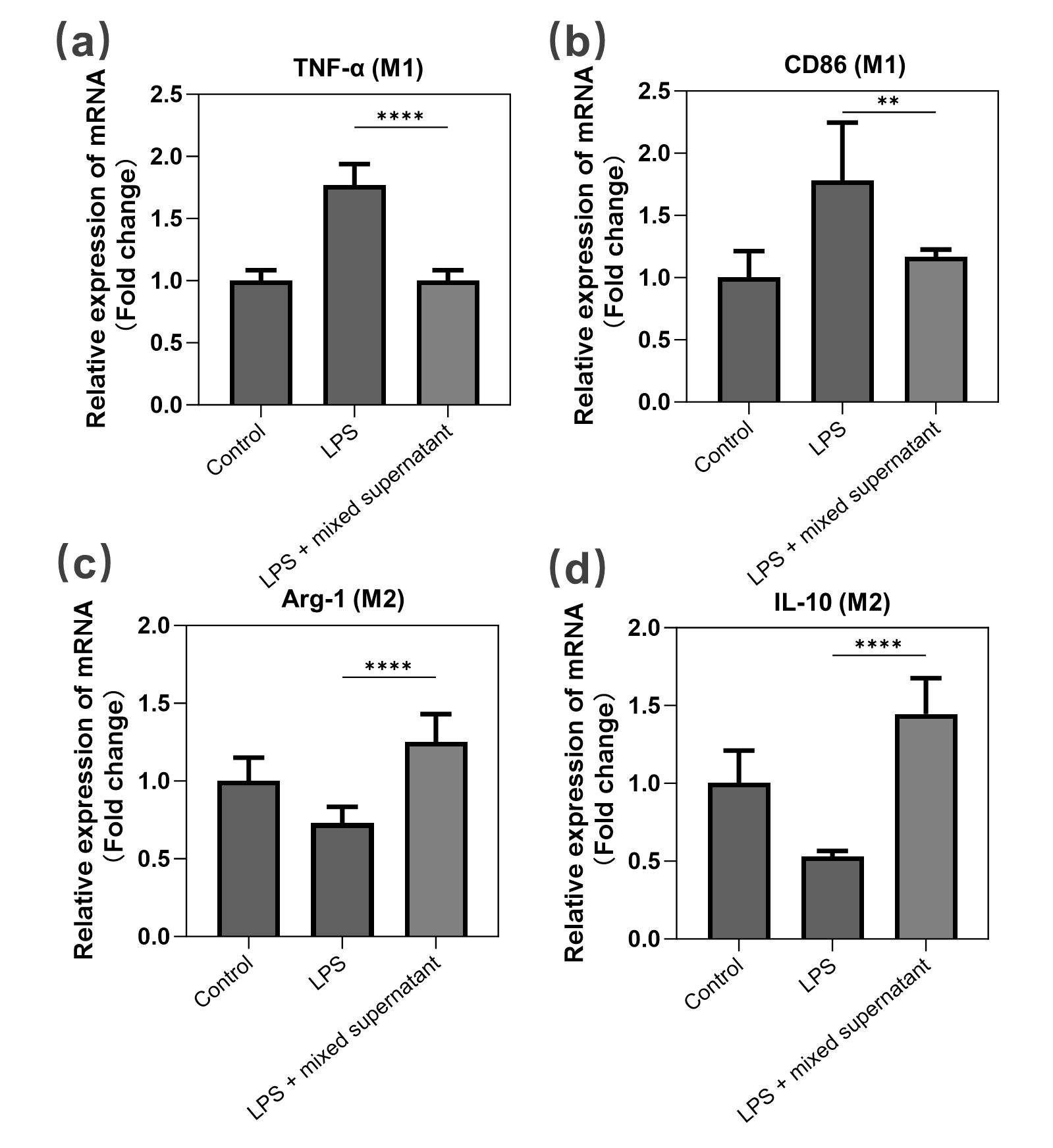


**Figure S29.** RT-qPCR analysis of macrophage polarization and inflammatory factor expression. Quantitative analysis of the relative mRNA expression levels for (a) TNF-α, (b) CD86, (c) Arg-1, and (d) IL-10 in RAW 264.7 macrophages. Cells were either pre-treated with 100 ng mL^-1^ Pg-LPS or not and then stimulated with supernatants collected from the *Bifidobacterium breve* and *Ligilactobacillus salivarius*. The data are presented as the mean ± SD of n = 3. The significance of the data was calculated by the one-way ANOVA. ns: no significance, **p* < 0.05, ***p* < 0.01, ****p* < 0.001, *****p* < 0.0001.


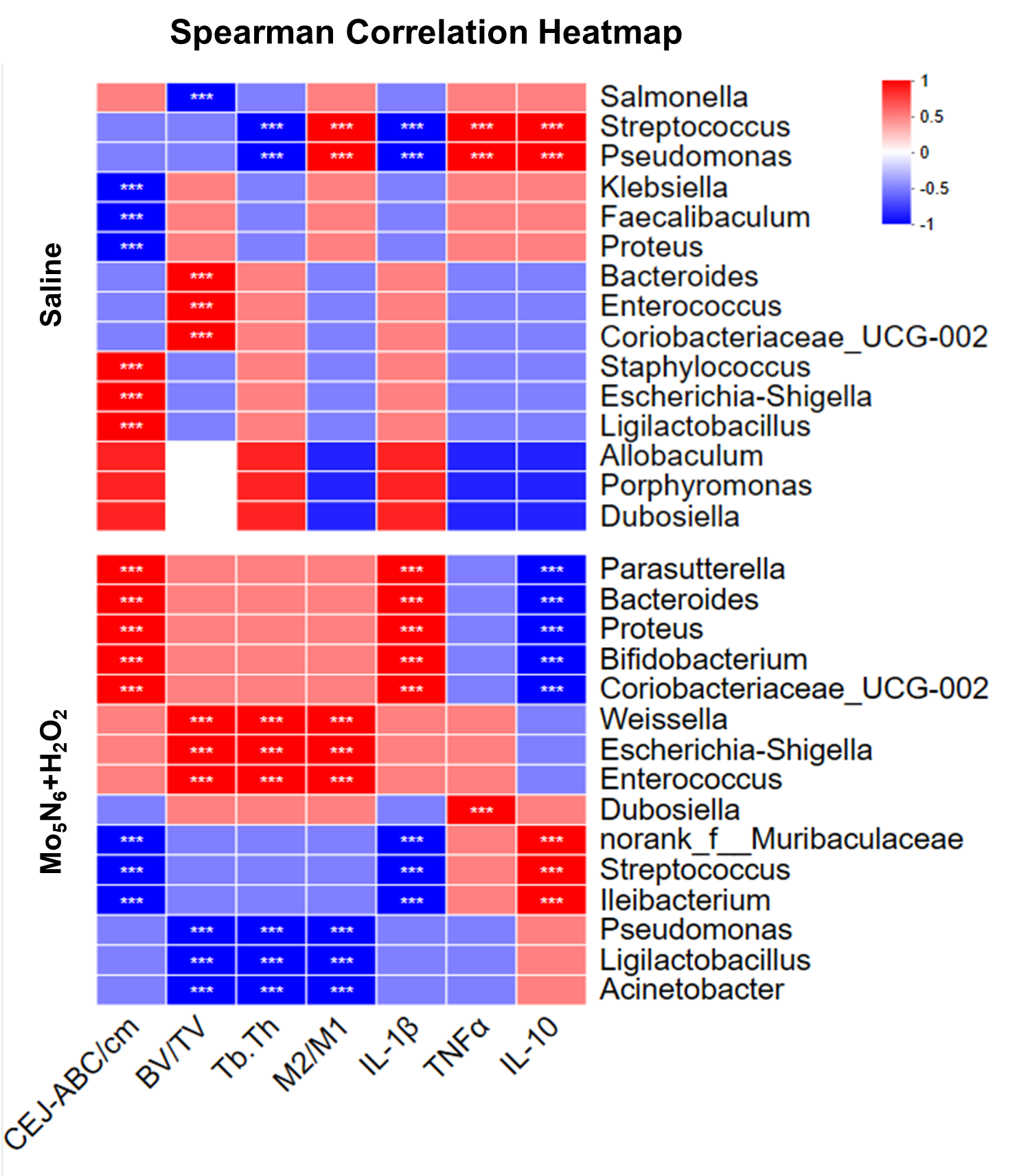


**Figure S30.** The Spearman correlation heatmap visually displays the relationships between the abundance of 15 genera and clinical parameters in the Saline group and the Mo_5_N_6_ + H_2_O_2_ group, providing an intuitive representation of both the strength and statistical significance of correlations between multiple clinical factors and different microbial taxa. In the figure, the X-axis and Y-axis represent the clinical parameters and microbial species, respectively. The R-values obtained from statistical calculations are represented by color gradients in the heatmap, while asterisks indicate statistical significance: **p* < 0.05, ***p* < 0.01, ****p* < 0.001, *****p* < 0.0001. The color scale on the right illustrates the range of R-values.


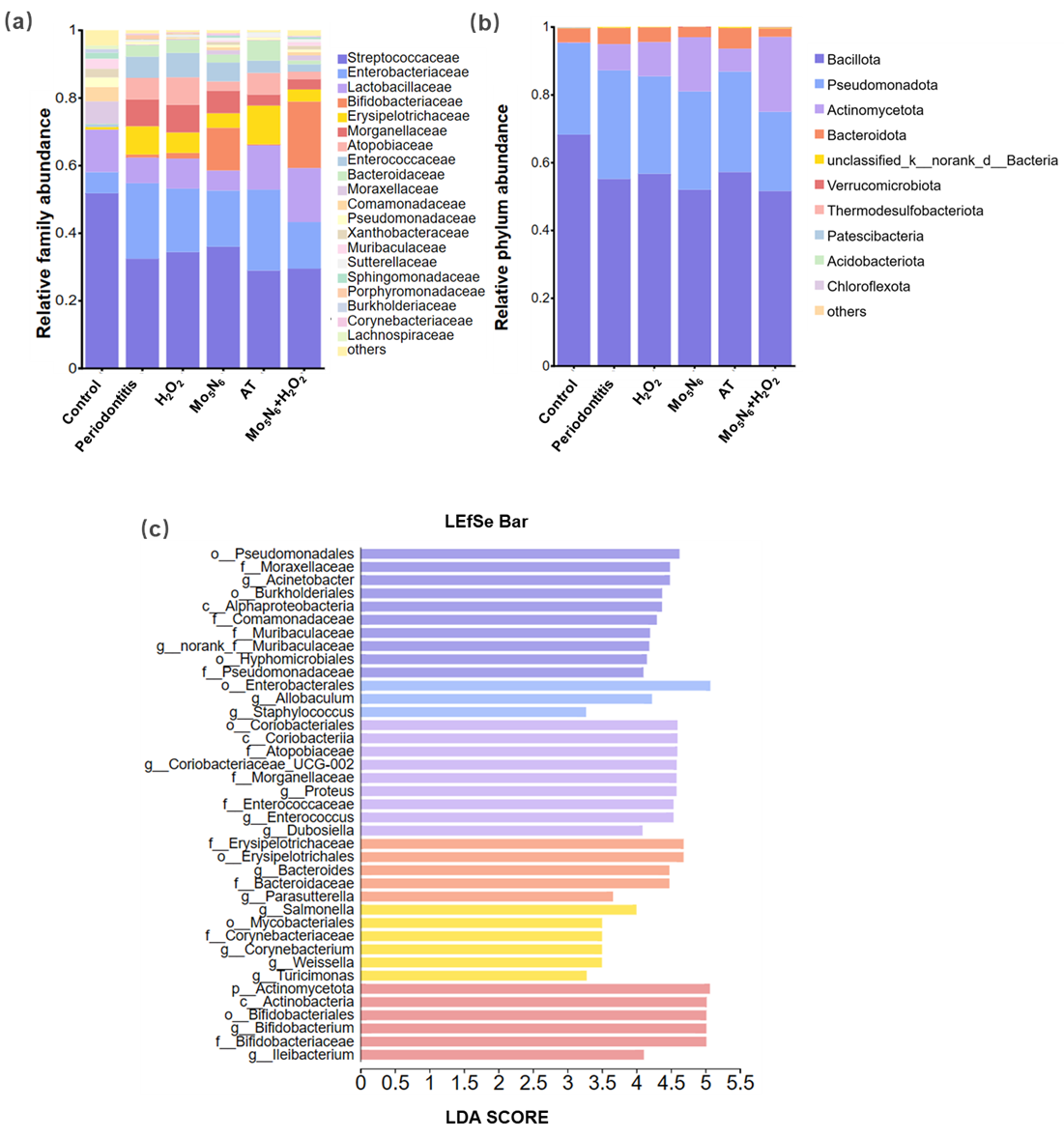


**Figure S31.** LEfSe analysis of subgingival microbiota (p, phylum; o, order; f, family; g, genus). Only the taxes having a linear discriminant analysis value >2 are shown in the figure.


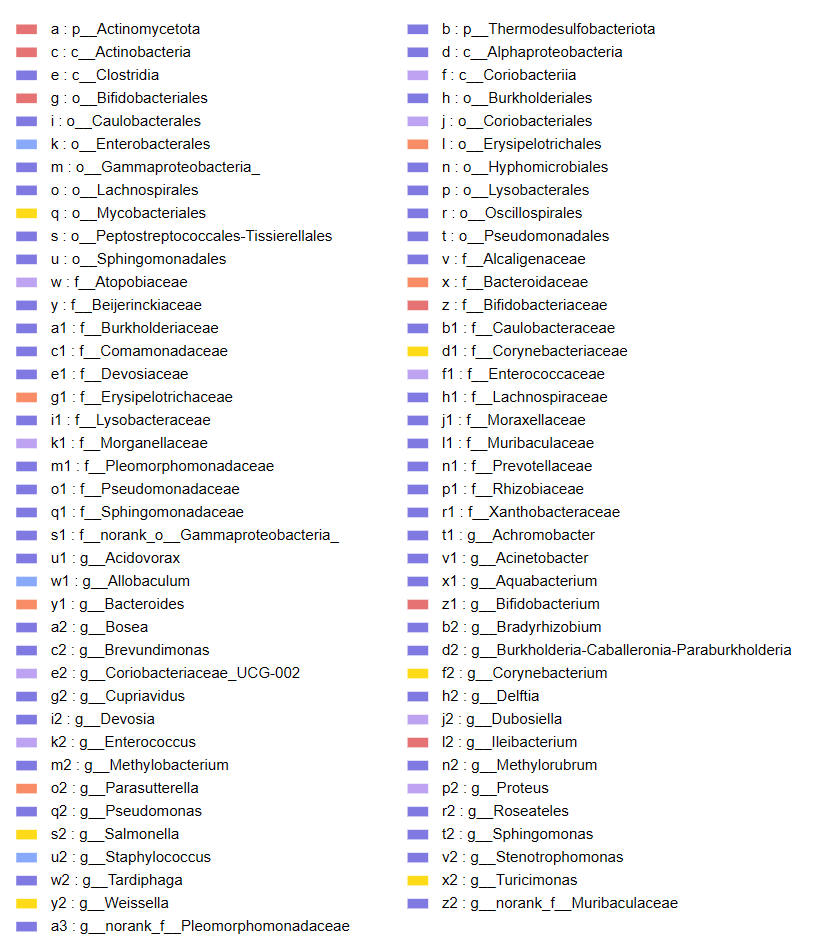


**Figure S32.** Legend of taxa from Circular Cladogram in LEfSe analysis in Figure 5n. Different colors and labels correspond to various microbial taxa from phylum to genus levels, interpreting the each branch in the cladogram.


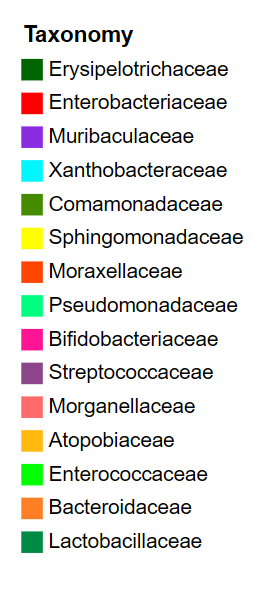


**Figure S33.** The "Taxonomy" section presents the classification at the family level of bacterial taxa shown in the heatmap in Figure 5o.


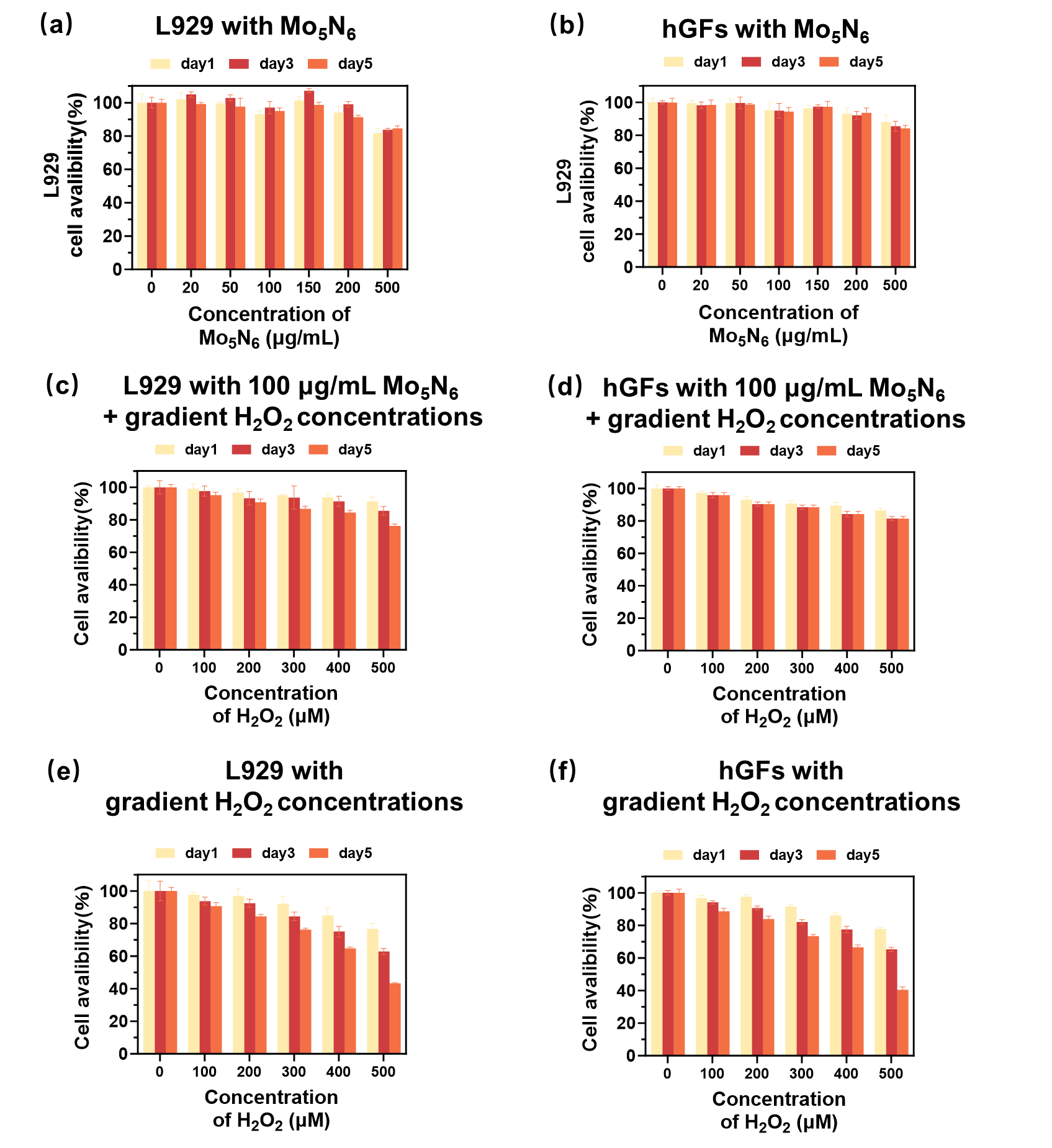


**Figure S34.** *In vitro* biocompatibility tests of Mo_5_N_6_ and H_2_O_2_ by CCK-8 assay. Effect of different concentrations of Mo_5_N_6_ treated for various times on (a) murine L929 cell viability and (b) human gingival fibroblast (hGFs) viability. Effect of 100 μg mL^-1^ Mo_5_N_6_ combined with different concentrations of H_2_O_2_ treated for various times on (c) murine L929 cell viability and (d) hGFs viability. Effect of different concentrations of H_2_O_2_ treated for various times on (e) murine L929 cell viability and (f) hGFs viability. The data are presented as the mean ± SD of n = 3. The significance of the data was calculated by the one-way ANOVA. ns: no significance, **p* < 0.05, ***p* < 0.01, ****p* < 0.001, *****p* < 0.0001.

**
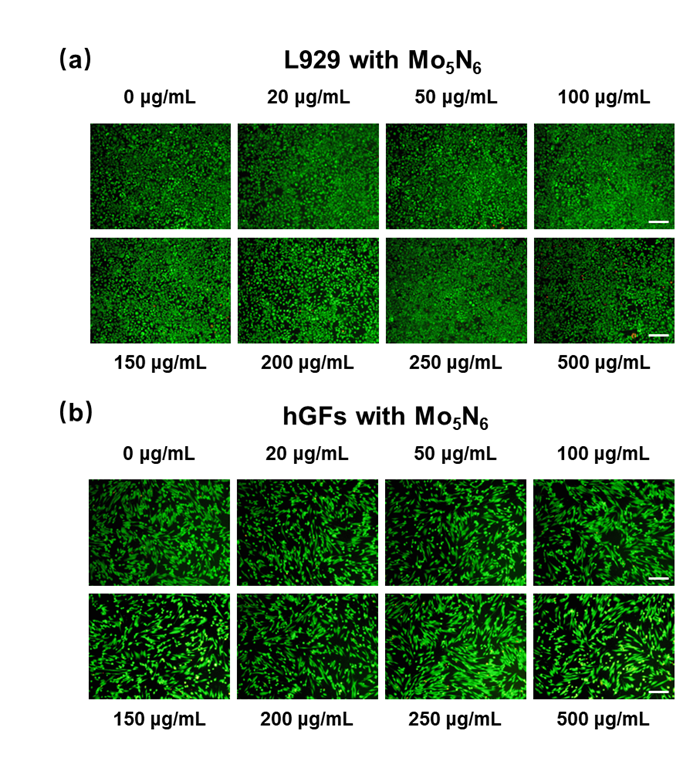
**

**Figure S35.** *In vitro* biocompatibility tests of Mo_5_N_6_ by Live/Dead staining. (a) Fluorescence images of Live/Dead staining of murine L929 cells under different concentrations of Mo_5_N_6_ for 48 hours. (b) Fluorescence staining images of human gingival fibroblasts (hGFs) under different concentrations of Mo_5_N_6_ for 48 hours. Scale bar: 200 μm, n = 3.


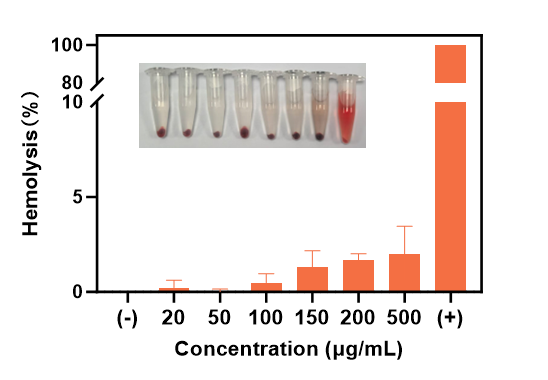


**Figure S36.** Hemolysis assay results treated by Mo_5_N_6_ at gradient concentrations (0-500 μg mL^-1^) for six hours. The data are presented as the mean ± SD of n = 3. The significance of the data was calculated by the one-way ANOVA. ns: no significance, **p* < 0.05, ***p* < 0.01, ****p* < 0.001, *****p* < 0.0001.


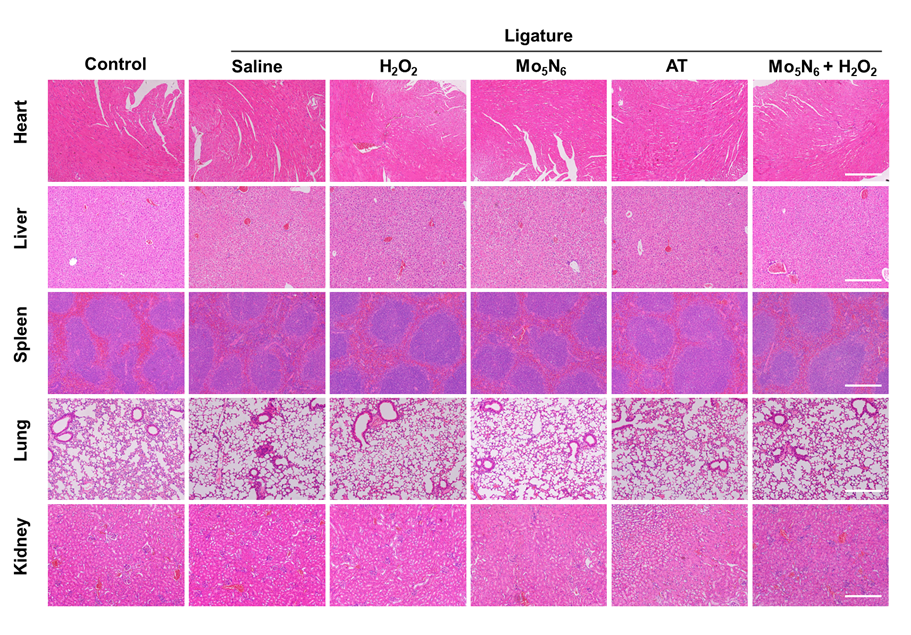


**Figure S37.** H&E-stained images of major organs from the Control/Saline/H_2_O_2_/Mo_5_N_6_/AT/Mo_5_N_6_ + H_2_O_2_ group in ligature-induced mice after treatment for 14 days. Scale bar: 200 μm, n = 3.

**Table S1. Comparison of Michaelis-Menten constants (*K*_m_) and maximum initial reaction rates (*V*_max_) of the catalytic reaction.** Reaction pH and temperature are listed when explicitly provided in the cited references. ‘-’ indicates not specified in the original paper.

| Nanozymes | pH | Temperature | *Km* (mM) | *V_max_* (M·s^-1^) | Ref. |
| --- | --- | --- | --- | --- | --- |
| Mo_5_N_6_ | 4.0 | 37 ^o^C | 0.075 | 12.64 × 10^-6^ | This work |
| HRP | 5.0 | 30 ^o^C | 3.72 | 8.7 × 10^-8^ | ^1^ |
| GCNT-Fe_3_O_4_ | 4.0 | 25 ^o^C | 2.52 | 0.387 × 10^-8^ | ^2^ |
| Carbon dots | - | - | 0.7468 | 1.086 × 10^-8^ | ^3^ |
| CuO_2_ | 6.0 | 37 ^o^C | 0.34 | 1.43 × 10^-8^ | ^4^ |
| Cu-hemin MOF | - | - | 0.075 | 1.26 × 10^-6^ | ^5^ |
| Cu-ZIF | - | - | 6.7 | 0.91 × 10^-8^ | ^6^ |
| Au-Pt nps | - | - | 1100 | 7.17 × 10^-7^ | ^7^ |
| AuNS | - | - | 721 | 4.9 × 10^-7^ | ^8^ |
| ZIF-67 nanosheets | - | - | 3.52 | 0.28 × 10^-8^ | ^9^ |
| Fe-g-C_3_N_4_ | - | - | 8.956 | 1.46 × 10^-8^ | ^10^ |
| Cur-AuNPs | - | - | 3.10 | 9.27 × 10⁻⁷ | ^11^ |

**Table S2. *K_m_* and *V_max_* collected from the cited literature under peroxidase-like catalytic conditions.** Due to differences in experimental protocols, substrates, and normalization methods among studies, the kinetic parameters are provided for qualitative comparison rather than strict quantitative ranking.

| Nanozymes | *Km* (mM) | *V_max_* (M·s^-1^) | Ref. |
| --- | --- | --- | --- |
| Mo_5_N_6_ | 0.075 | 12.64 × 10^-6^ | This work |
| Mo_SA_-N_3_-C | 2 | 3.7 × 10^-7^ | ^12^ |
| MoN_x_O_y_ | 0.18 | 9.37 × 10^-7^ | ^13^ |
| M-MoS_2_ | 0.40 | 2.01 × 10^-7^ | ^14^ |
| N-doped MoS_2_ | 0.45 | 4.35 × 10^-8^ | ^15^ |
| ZnN_4_ SAEs | 40.16 | 1.22 × 10^-7^ | ^16^ |
| TiN NB | 9.27 | 5.56 × 10^-8^ | ^17^ |
| Fe-N_4_-C | 175.5 | 5.68 × 10^-7^ | ^18^ |
| MoO_3_ | 0.22 | 3.75 × 10^-8^ | ^19^ |

**Table S3. Primers used for qRT-PCR**

| Gene |  | Primer sequence (5’-3’) |
| --- | --- | --- |
| *β-Actin (Mouse)* | Forward | GGCTGTATTCCCCTCCATCG |
|  | Reverse | CCAGTTGGTAACAATGCCATGT |
| *TNF-α (Mouse)* | Forward | CCCTCACACTCAGATCATCTTCT |
|  | Reverse | GCTACGACGTGGGCTACAG |
| *CD86 (Mouse)* | Forward | TGTTTCCGTGGAGACGCAAG |
|  | Reverse | TTGAGCCTTTGTAAATGGGCA |
| *Arg-1 (Mouse)* | Forward | CCACAGTCTGGCAGTTGGAAG |
|  | Reverse | GGTTGTCAGGGGAGTGTTGATG |
| *IL-10 (Mouse)* | Forward | GCTCTTACTGACTGGCATGAG |
|  | Reverse | CGCAGCTCTAGGAGCATGTG |

**Reference**

(1) Liu, P.; Shi, C.; Liu, Y.; Yang, F.; Yang, Y. Development of AI-integrated smartphone sponge-based sensors utilizing His@Co-NC nanozymes for highly sensitive sarcosine detection. *Biosens Bioelectron* **2025**, *286*, 117621.

(2) Yousefinejad, S.; Rasti, H.; Hajebi, M.; Kowsari, M.; Sadravi, S.; Honarasa, F. Design of C-dots/Fe_3_O_4_ magnetic nanocomposite as an efficient new nanozyme and its application for determination of H_2_O_2_ in nanomolar level. *Sensors and Actuators B: Chemical* **2017**, *247*, 691-696.

(3) Li, Y.; Gu, X.; Zhao, J.; Xi, F. Fabrication of a Ratiometric Fluorescence Sensor Based on Carbon Dots as Both Luminophores and Nanozymes for the Sensitive Detection of Hydrogen Peroxide. *Molecules* **2022**, *27*, 7379.

(4) Li, J.; Yi, W.; Luo, Y.; Yang, K.; He, L.; Xu, C.; Deng, L.; He, D. GSH-depleting and H_2_O_2_-self-supplying hybrid nanozymes for intensive catalytic antibacterial therapy by photothermal-augmented co-catalysis. *Acta Biomater* **2023**, *155*, 588-600.

(5) Liu, F.; He, J.; Zeng, M.; Hao, J.; Guo, Q.; Song, Y.; Wang, L. Cu–hemin metal-organic frameworks with peroxidase-like activity as peroxidase mimics for colorimetric sensing of glucose. *Journal of Nanoparticle Research* **2016**, *18*, 106.

(6) Mechoor, A.; Berchmans, S.; Venkatachalam, G. Bimetallic Cu–Zn Zeolitic Imidazolate Frameworks as Peroxidase Mimics for the Detection of Hydrogen Peroxide: Electrochemical and Spectrophotometric Evaluation. *ACS Omega* **2023**, *8*, 39636-39650.

(7) Gao, Y.-C.; Wang, C.; Zhang, C.-X.; Li, H.-W.; Wu, Y. Controlled preparation and application of glutathione capped gold and platinum alloy nanoclusters with high peroxidase-like activity. *Journal of Materials Science & Technology* **2022**, *109*, 140-146.

(8) Fernández-Lodeiro, C.; Fernández-Lodeiro, J.; Fernández-Lodeiro, A.; Nuti, S.; Lodeiro, C.; LaGrow, A.; Pérez-Juste, I.; Pérez-Juste, J.; Pastoriza-Santos, I. Synthesis of tuneable gold nanostars: the role of adenosine monophosphate. *Journal of Materials Chemistry C* **2023**, *11*, 12626-12636.

(9) Zhang, W.; Li, X.; Cui, T.; Li, S.; Qian, Y.; Yue, Y.; Zhong, W.; Xu, B.; Yue, W. PtS_2_ nanosheets as a peroxidase-mimicking nanozyme for colorimetric determination of hydrogen peroxide and glucose. *Mikrochim Acta* **2021**, *188*, 174.

(10) Tian, J.; Liu, Q.; Asiri, A. M.; Qusti, A. H.; Al-Youbi, A. O.; Sun, X. Ultrathin graphitic carbon nitride nanosheets: a novel peroxidase mimetic, Fe doping-mediated catalytic performance enhancement and application to rapid, highly sensitive optical detection of glucose. *Nanoscale* **2013**, *5*, 11604-11609.

(11) Sudhesh, P.; Sruthi, S.; Jose, M.; Vyshnavi, K.; Aiswarya, P.; Manu, R. Naked eye detection of hydrogen peroxide via curcumin functionalised gold nanoparticles. *Sci Rep* **2025**, *15*, 16896.

(12) Wang, Y.; Jia, G.; Cui, X.; Zhao, X.; Zhang, Q.; Gu, L.; Zheng, L.; Li, L. H.; Wu, Q.; Singh, D. J.; et al. Coordination Number Regulation of Molybdenum Single-Atom Nanozyme Peroxidase-like Specificity. *Chem* **2021**, *7*, 436-449.

(13) Zhang, X.; Lin, P.; Wu, Y.; Fan, M.; Xia, C.; Guo, L. Thermophilic MoN_x_O_y_ nanozyme for highly efficient degradation of phenolic pollutants. *Commun Chem* **2025**, *8*, 380.

(14) Wang, L.; Li, B.; You, Z.; Wang, A.; Chen, X.; Song, G.; Yang, L.; Chen, D.; Yu, X.; Liu, J.; et al. Heterojunction of Vertically Arrayed MoS_2_ Nanosheet/N-Doped Reduced Graphene Oxide Enabling a Nanozyme for Sensitive Biomolecule Monitoring. *Anal Chem* **2021**, *93*, 11123-11132.

(15) Feng, L.; Zhang, L.; Zhang, S.; Chen, X.; Li, P.; Gao, Y.; Xie, S.; Zhang, A.; Wang, H. Plasma-Assisted Controllable Doping of Nitrogen into MoS_2_ Nanosheets as Efficient Nanozymes with Enhanced Peroxidase-Like Catalysis Activity. *ACS Appl Mater Interfaces* **2020**, *12*, 17547-17556.

(16) Xu, B.; Wang, H.; Wang, W.; Gao, L.; Li, S.; Pan, X.; Wang, H.; Yang, H.; Meng, X.; Wu, Q.; et al. A Single-Atom Nanozyme for Wound Disinfection Applications. *Angew Chem Int Ed Engl* **2019**, *58*, 4911-4916.

(17) Liu, J.; Wang, A.; Liu, S.; Yang, R.; Wang, L.; Gao, F.; Zhou, H.; Yu, X.; Liu, J.; Chen, C. A Titanium Nitride Nanozyme for pH-Responsive and Irradiation-Enhanced Cascade-Catalytic Tumor Therapy. *Angew Chem Int Ed Engl* **2021**, *60*, 25328-25338.

(18) Xi, J.; Zhang, R.; Wang, L.; Xu, W.; Liang, Q.; Li, J.; Jiang, J.; Yang, Y.; Yan, X.; Fan, K.; et al. A Nanozyme‐Based Artificial Peroxisome Ameliorates Hyperuricemia and Ischemic Stroke. *Advanced Functional Materials* **2020**, *31*.

(19) Niu, Y.; Ji, T.; Yang, R.; Chen, B.; Zheng, Z.; Li, M.; Wang, S.; Li, Z.; Li, Z.; Tang, W. Portable colorimetric detection of copper based on enhanced peroxidase-like activity of MoO_3_ nanobelts. *Anal Methods* **2025**, *17*, 9191-9199.
